# Comparative transcriptome analysis of T lymphocyte subpopulations and identification of critical regulators defining porcine thymocyte identity

**DOI:** 10.3389/fimmu.2024.1339787

**Published:** 2024-02-07

**Authors:** Pingping Han, Wei Zhang, Daoyuan Wang, Yalan Wu, Xinyun Li, Shuhong Zhao, Mengjin Zhu

**Affiliations:** ^1^ Key Lab of Agricultural Animal Genetics, Breeding, and Reproduction of Ministry of Education, Huazhong Agricultural University, Wuhan, China; ^2^ The Cooperative Innovation Center for Sustainable Pig Production, Huazhong Agricultural University, Wuhan, China

**Keywords:** comparative transcriptome, T cell development, co-expression analysis, gene regulatory network, transcription factor, GWAS

## Abstract

**Introduction:**

The development and migration of T cells in the thymus and peripheral tissues are crucial for maintaining adaptive immunity in mammals. However, the regulatory mechanisms underlying T cell development and thymocyte identity formation in pigs remain largely underexplored.

**Method:**

Here, by integrating bulk and single-cell RNA-sequencing data, we investigated regulatory signatures of porcine thymus and lymph node T cells.

**Results:**

The comparison of T cell subpopulations derived from porcine thymus and lymph nodes revealed that their transcriptomic differences were influenced more by tissue origin than by T cell phenotypes, and that lymph node cells exhibited greater transcriptional diversity than thymocytes. Through weighted gene co-expression network analysis (WGCNA), we identified the key modules and candidate hub genes regulating the heterogeneity of T cell subpopulations. Further, we integrated the porcine thymocyte dataset with peripheral blood mononuclear cell (PBMC) dataset to systematically compare transcriptomic differences between T cell types from different tissues. Based on single-cell datasets, we further identified the key transcription factors (TFs) responsible for maintaining porcine thymocyte identity and unveiled that these TFs coordinately regulated the entire T cell development process. Finally, we performed GWAS of cell type-specific differentially expressed genes (DEGs) and 30 complex traits, and found that the DEGs in thymus-related and peripheral blood-related cell types, especially CD4_SP cluster and CD8-related cluster, were significantly associated with pig productive and reproductive traits.

**Discussion:**

Our findings provide an insight into T cell development and lay a foundation for further exploring the porcine immune system and genetic mechanisms underlying complex traits in pigs.

## Introduction

T lymphocytes, as a major component of the adaptive immune system, play an essential role in eliminating invading pathogens, maintaining self-tolerance, and enhancing anti-tumor immunity (1). The thymus provides a site for T cell differentiation, development, and maturation, and these processes are co-regulated by T cells and thymic epithelial cells. Specifically, hematopoietic progenitor cells or thymus-seeding progenitor cells are originated from the bone marrow or fetal liver, entering the thymus via the blood circulation, where they further differentiate into thymic progenitor cells (2). Early thymic progenitor cells, initially lacking the expression of CD4 and CD8, are referred to as double-negative (DN) thymocytes, and subsequently they acquire CD4 and CD8 co-receptors, advancing to the double-positive (DP) stage (3). DP thymocytes that successfully express functional αβ T cell receptor (TCR) undergo positive selection mediated by cortical thymic epithelial cells (cTECs) and negative selection mediated by medullary thymic epithelial cells (mTECs), ultimately differentiating into either CD4 or CD8 single-positive (SP) thymocytes (4–6). After acquiring self-MHC-restriction and non-autoreactivity, naive T cells migrate to peripheral lymphoid tissues through the blood circulation, where they wait for activation and subsequent immune responses.

Mammalian T cell development is a complex and dynamic process. Currently, our understanding of T cell differentiation and migration is mainly based on the evidence from humans and mice, but the related knowledge of T cell differentiation and migration in pigs remains limited. Since pigs are the most important meat-producing livestock breed globally, a profound understanding of their immune system is crucial for improving their overall health and production efficiency. The high similarity of pigs to humans in anatomy, genetics, and physiology makes them an increasingly popular large animal model in clinical research. As a biomedical model, pigs own a human-like immune system, but they differ from mice and human in several immune characteristics (7). For instance, pig is recognized as a species with a large proportion of γδ T cells, while humans and mice have only a small proportion of these cells (8). In addition, the existing comparative transcriptome studies have focused on flow-sorted cell populations from porcine peripheral blood, including DP, CD4+, and CD8+ T cells (9–11). However, the information on immune tissues other than blood, such as mesenteric lymph node, remains scarce. The transcriptomic differences between thymic T cells and peripheral T cells in pigs have not been investigated so far. Therefore, it is necessary to investigate the phenotypic and functional characteristics of T cell subpopulations in different immune tissues in pigs.

At present, bulk RNA-seq methods and microarray technologies for revealing T cell development have advanced (11, 12). However, these technologies tend to examine only the average transcriptional signature of preselected cell types since whole tissue rather than individual cells are investigated. In contrast, single-cell RNA-seq sequencing (scRNA-seq) technology can simultaneously analyze the transcriptomes of hundreds to thousands of individual cells, thus making it possible to dissect cellular heterogeneity, identify cell types, and characterize developmental dynamics. In recent years, scRNA-seq has been used to map the cell atlas of porcine organs and tissues, including the brain (13), lung (14), ileum (15), testis (16) and peripheral blood (11). Using scRNA-seq, Gu et al. (17) have uncovered the cellular heterogeneity and developmental dynamics of porcine thymus. Thymus is a highly specialized organ of the immune system. However, the mechanisms underlying specific phenotype maintenance during thymic T cell development remain unclear. Transcription factors (TFs)-mediated gene regulatory networks are considered important for determining cell type identify (18, 19). With the accumulation of massive single-cell data, many efficient and feasible methods such as single-cell regulatory network inference and clustering (SCENIC) have been established to identify TFs maintaining cell identity (20).

In this study, we first performed a cross-tissue cross-cell type transcriptome comparison of 7 T cell subpopulations classified according to cell surface markers CD3, CD4, and CD8 from porcine thymus and lymph nodes. We integrated bulk RNA-seq data with recently released scRNA-seq data of thymic samples to identify thymocyte heterogeneity and the TFs controlling lineage differentiation. In addition, we integrated 8 peripheral blood mononuclear cell (PBMC) datasets (including 7 previously published datasets and 1 dataset generated in our laboratory) with the porcine thymocyte dataset to compare the transcriptomic differences of T cell types between peripheral blood and thymus. Finally, trait-related cell types were identified by combining cell type-specific differentially expressed genes (DEGs) with GWAS signals of 30 complex traits in pigs. To our knowledge, this study elucidated gene regulatory signatures of T cell lineage differentiation in the porcine thymus for the first time, thereby extending our understanding of cellular heterogeneity, transcriptional networks, and immune system in pigs.

## Materials and methods

### Animals

All experimental procedures were approved by the Institutional Animal Care and Use Committee of Huazhong Agricultural University, China. The samples used for the comparative transcriptome experiment were derived from three 3-day-old healthy Large White pigs from the experimental farm of Huazhong Agricultural University (Wuhan, China).

### Cell suspension preparation

Fresh thymic and mesenteric lymph node tissues were obtained from 3 pigs and were washed with cold phosphate-buffered saline (PBS). Subsequently, the thymic and mesenteric lymph node tissues were minced and digested with 1mg/mL and 2 mg/mL collagenase I for 1 h at 37°C, respectively. Equal volumes of 5% fetal bovine serum were added to terminate the digestion reaction. The dissociated cells were filtered through a 100 µm cell strainer, centrifuged at 1000 rpm for 10 min at 4°C, and diluted to 1×10^6^/mL. The diluted cells were counted using trypan blue staining.

### Fluorescence-activated cell sorting and antibodies used for it

**Figure 1:**
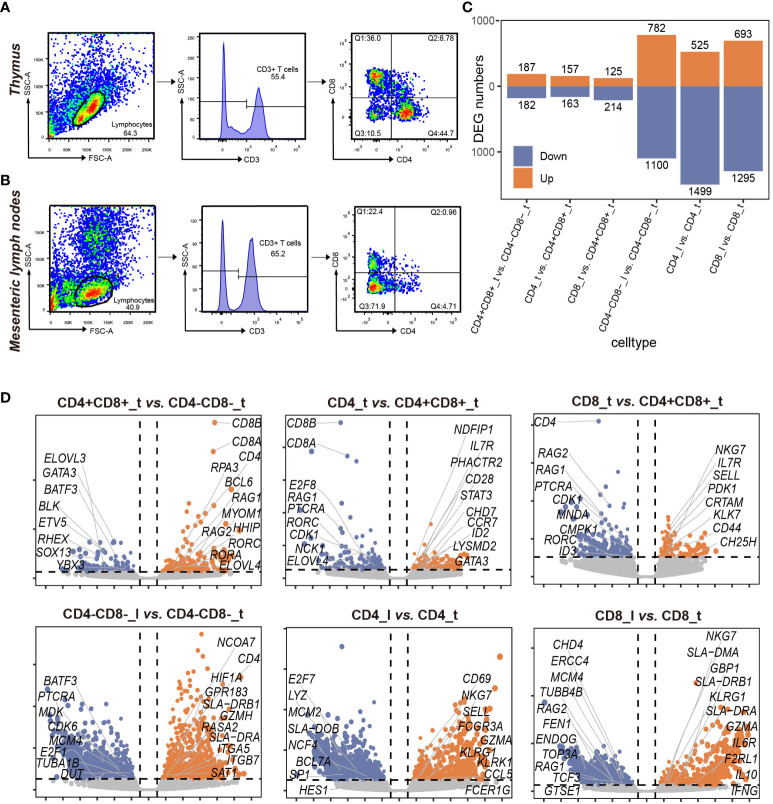
Sorting and comparative transcriptome analysis of 7 T cell subpopulations in porcine thymus and lymph nodes. **(A)** Porcine thymus 4 T cell subpopulations obtained by fluorescence-activated cell sorting (FACS). Lymphocytes obtained from porcine thymus samples based on flow cytometry forward scatter (FSC) and side scatter (SSC) (left). Histogram of the percentage of viable CD3+ cells identified from porcine lymphocytes using the flow cytometry gating strategy (middle). 7-AAD was used to label dead cells. CD3+ cells were further divided into 4 populations based on CD4 and CD8 fluorescence intensity: CD4-CD8+ (Q1), CD4+CD8+ (Q2), CD4-CD8- (Q3), and CD4+CD8- (Q4) T cells (right). **(B)** 3 T cell subpopulations obtained by fluorescence-activated cell sorting (FACS) in porcine lymph nodes. Lymphocytes obtained from porcine lymph node samples based on flow cytometry forward scatter (FSC) and side scatter (SSC) (left). Histogram of the percentage of viable CD3+ cells identified from porcine lymphocytes using the flow cytometry gating strategy (middle). 7-AAD was used to label dead cells. CD3+ cells were further divided into 3 populations based on CD4 and CD8 fluorescence intensity: CD4-CD8+ (Q1), CD4-CD8- (Q3), and CD4+CD8- (Q4) T cells (right). **(C)** Number of differentially expressed genes (DEGs) in 7 T cell subpopulations from thymus and lymph nodes. The experiments were performed with 3 biological replicates for each T cell population. Orange and blue denote up-regulated and down-regulated DEGs, respectively. **(D)** Volcano plot of the DEGs (|log_2_FC| > 1 and *P*-value < 0.05) in pairwise comparisons of the indicated T cell subpopulations (n=3). Orange and blue dots denote up-regulated and down-regulated DEGs, respectively.

Fluorescence-activated cell sorting was performed at the School of Life Science and Technology, Huazhong Agricultural University. The antibodies used for flow cytometry included FITC-conjugated mouse anti-pig CD3ϵ (clone BB23-8E6-8C8, isotype IgG2a, κ; BD Pharmingen), PE-conjugated mouse anti-pig CD4 monoclonal antibody (clone 74-12-4, isotype IgG2b, κ; BD Pharmingen), and APC-conjugated mouse anti-pig CD8α monoclonal antibody (clone 76-2-11, isotype IgG2a, κ; BD Pharmingen). In the experimental group, 1 mL of porcine thymocyte suspension was transferred to a 1.5 mL RNAase-free EP tube and added with 3 μL of CD3ϵ, 6 μL of CD4, and 6 μL of CD8α antibodies. The 0.5 mL thymocyte suspension was transferred to 1.5 mL RNase-free EP tubes, added with 1.5 μL of CD3ϵ, 3 μL of CD4, or 3 μL of CD8α antibodies respectively, and used as the three control groups. The thymocyte suspension without any addition was used as a blank control. All the cells were incubated at 4°C for 30 min in the dark, and added with 10 μL 7-amino-actinomycin D (7-AAD, Viaprobe, BD Pharmingen) before flow sorting to remove dead cells. Porcine thymocytes were initially divided into CD3-positive and CD3-negative fractions using FACS. The CD3-positive fraction was subjected to FACS gating based on forward scatter (FSC) and side scatter (SSC) parameters, further divided into 3 distinct populations according to CD4 and/or CD8 marker expression, namely, CD4-CD8+ (Q1), CD4+CD8+ (Q2), and CD4+CD8- (Q4) T cells. The CD4-CD8- (Q3) cell population was enriched through negative sorting (**Figure 1A**). Using the above-mentioned sorting strategy, we divided porcine mesenteric lymph node cells into 3 cell populations including CD4-CD8- (Q3), CD4+CD8- (Q4), and CD4-CD8+ (Q1) T cells (**Figure 1B**). The purity of each T cell population exceeded 90%. All data were processed using the FlowJo v7.6.1 software (TreeStar Inc., San Carlos, CA, USA).

### Total RNA extraction, library construction, and RNA-seq sequencing

A total of 1,000 cells with no less than 1 μg total RNA was extracted from each T cell population using a RNeasy Mini kit (Qiagen, Valencia, CA, USA) according to the manufacturer’s protocol with three biological replicates. RNA purity and concentration were determined using a NanoPhotometer® spectrophotometer (IMPLEN, CA, USA). The cDNA library construction and sequencing were carried out in accordance with the Illumina standard protocol by Beijing Novogene Bioinformatics Technology Company. The library quality was evaluated using an Agilent Bioanalyzer 2100 system. The DNA library was sequenced on an Illumina Hiseq platform, and 150bp paired-end reads were generated. The experiments were conducted with three independent biological replicates for each T cell population.

### Bulk RNA-seq data processing

Using in-house Perl script, quality control of the raw FASTQ data was performed to remove adaptor sequences, reads with ploy-N, low-quality reads, and clean reads were obtained for subsequent analysis (21). The porcine reference genome (Sscrofa 11.1) and gene annotation files (v11.1.98) were downloaded from the Ensemble website, and gene annotation files were modified, as previously described (22). An updated complete list of gene names was provided in **Supplementary Table 1**. We utilized STAR (v2.7.5a) to build reference genome index files and align paired-end clean reads to the reference genome. Next, gene quantification was performed using RSEM (v1.2.31). The raw counts, FPKM (fragments per kilobase of transcripts per million mapped fragments) values and TPM (transcripts per million) values of each gene or isoform were contained in the output files. Differentially expressed genes (DEGs) were identified using the DESeq2 package (v1.34.0) with the thresholds of *P*-value < 0.05 and |log_2_ fold change (FC)| > 1. The volcano plot and heatmap of DEGs were drawn using the ggplot2 (v3.4.2) and pheatmap (v1.0.12) packages, respectively. To further investigate the function of each T cell subpopulation from the thymus and lymph nodes, we performed GO enrichment analysis of up-regulated DEGs from pairwise comparisons at the Metascape website (https://metascape.org/) with default parameters (23).

### Weighted co-expression network construction

A weighted gene co-expression network was constructed based on the TPM data matrix using the WGCNA package (v1.72.1) (24). Before WGCNA, genes with low expression values were filtered. Samples were clustered using the “hclust” function, and outlier samples were removed. The “pickSoftThreshold” function was used to select the optimal soft threshold to ensure the scale-free distribution of network. Next, the “blockwiseModules” function was applied to construct network and identify module. Each module consisted of at least 30 genes, and gene modules with similarity > 75% were merged automatically. The correlation between gene modules and 7 cell subpopulations was investigated through Pearson correlation analysis and visualized with the “labeledHeatmap” function. We further screened key modules most associated with specific subpopulations based on correlation and *P*-value.

### Screening of hub genes

The hub genes in key module were identified by calculating the gene significance (GS) and module membership (MM). The GS refers to the correlation between the gene and the trait, while the MM represents the correlation between the module eigengene and the gene expression profile. Hub genes were screened with the cut-off criteria of GS > 0.5 and MM > 0.85. We defined the overlapping genes of DEGs obtained from bulk RNA-seq analysis and hub genes in key modules most related to T cell subpopulation as hub DEGs. We extracted the edges and nodes from the network with a threshold of 0.15 based on the weighted topological overlap matrix (TOM) using the “exportNetworkToCytoscape” function of WGCNA. Finally, Cytoscape software (v3.9.1) was utilized for network visualization, and the Maximal Clique Centrality (MCC) topology algorithm in Cytoscape’s CytoHubba plugin was used to identify important genes in a given network (25, 26).

### ScRNA−seq data processing

The raw gene expression matrix of porcine thymus (containing 2 samples) used in this study was downloaded from the GEO database (GSE192520), and scRNA-seq data were processed using the Seurat package (v4.3.0.1), as previously described (17). After removing the genes with low detection rates (expressed in less than 3 cells) and the cells in which gene number was < 200 or > 5,000 and mitochondrial ratio was > 11%, a total of 5,999 cells were obtained for subsequent analysis. After filtration, the gene counts in each cell were normalized using the “NormalizedData” function in Seurat package, and then cell cycle effects were regressed using the “ScaleData” function in this package. Afterwards, principal component analysis (PCA) was performed using the “RunPCA” function, and top 19 PCs (dim = 1:19) were selected for dimensionality reduction based on the “Elbowplot” function in Seurat package. Next, the main cell clusters were identified by the “FindClusters” function (resolution = 2.3) and visualized using uniform manifold approximation and projection (UMAP). The cell clusters were annotated using the conventional markers. We further manually merged some clusters with similar overlapping gene profiles. We also calculated the proportions of each cell type and visualized these cell types using the ggplot2 package. DEGs were identified in each cell type using the “FindAllMarkers” function (only.pos = TRUE, min.pct = 0.25, logfc.threshold = 0.25) with Wilcoxon rank sum test. We utilized the biomaRt package (v2.49.4) to convert porcine gene symbols into human homologs due to the limited availability of pig resources. GO enrichment analysis was performed at the Metascape website with default parameters.

### Gene set generation and gene set enrichment analysis

To investigate the consistency of bulk RNA-seq and scRNA-seq results, we conducted gene set enrichment analysis using previously described method (11). The unqualified samples and genes with extremely low expression levels (gene counts < 2 in one cell subpopulation) were filtered. As a result, a total of 13,245 qualified genes were obtained from 19 samples, which were subjected to differential gene expression analysis using DESeq2 package. A gene was defined as cell type-enriched gene if its expression level (mean of replicates) in a certain cell type was at least 2 folds as high as the mean gene expression level across all the remaining cell types, and the “results” function in DESeq2 package was used to identify cell type-enriched genes. Subsequently, we extracted the top 5%, 10%, 15%, 20%, 25%, and 30% of cell type-enriched genes from the porcine thymus bulk RNA-seq cell populations based on log_2_FC values to generate a list containing all highly enriched gene (HEG) sets.

Enrichment of gene set in porcine thymus scRNA-seq data was performed using AUCell package (v1.16.0). We extracted raw gene counts matrix from porcine thymus scRNA-seq data. The “AUCell_buildRankings” function was used to calculate gene rankings in each cell. Subsequently, the HEG set list file obtained from porcine thymus bulk RNA-seq populations and gene rankings were input to the “AUCell_calcAUC” function (with aucMaxRank set as top 5% of expressed genes) to calculate the area under the curve (AUC) score for each gene set in each cell. To map the AUC scores onto the UMAP plot coordinates of the scRNA-seq data, we manually set a threshold for each gene set based on the AUC score distribution using the “AUCell_plotHist” function. Finally, we calculated the average scaled AUC score for each cell cluster and visualized it using a heatmap.

### scRNA-seq analysis of merged porcine thymus and PBMC data

In this study, we integrated 7 previously published PBMC datasets (PRJEB43826) and 1 PBMC dataset generated in our laboratory (GSE247126). PBMC scRNA-seq data were pre-processed, as described by Herrera-Uribe et al. (11). Low-quality genes and cells were excluded from each dataset before integration. The “merge” function in Seurat was utilized to merge thymus and PBMC datasets. Subsequently, the “SelectIntegrationFeatures” function was employed to identify the genes with consistent expression pattern across the datasets. The “FindIntegrationAnchors” function was used to determine a set of anchors between the thymus and PBMC datasets. Next, an integrated dataset was created using the “IntegrateData” function. Then, the cluster analysis was performed using “RunPCA”, “FindNeighbours”. Finally, the “FindClusters” function was used to identify clusters (resolution = 1.4), and the “RunUMAP” function was used for visualization (reduction = “pca”, dims = 1:20).

Differential gene expression analysis was performed using the Wilcoxon rank sum test with the FindMarkers function in Seurat, as described by Ammons et al. (27). DEGs were identified with the thresholds of adjusted *P* < 0.01 and a |log_2_FC| > 0.58. Further, we performed GO enrichment analysis of up- and down-regulated DEGs at the Metascape website with default parameters.

### Pseudotime trajectory analysis of porcine thymocytes

We inferred the developmental trajectory of porcine thymocytes using Slingshot (v2.1.1) which was widely used in single-cell transcriptomics, and mapped the inferred trajectories onto UMAP for visualization (28). Additionally, we verified the consistency between our inferred developmental trajectory of porcine thymocytes and that constructed by Monocle3 package based on published scRNA-seq data in previous study (17).

### Single-cell regulatory network inference of porcine thymus

We conducted single-cell regulatory network analysis for each major cell type identified based on scRNA-seq data using SCENIC (v1.3.1) package, as previously described (20). Briefly, GENIE3 (v1.16.0) was applied to infer gene regulatory networks. RcisTarget (v1.14.0) was used to identify potential regulons based on DNA-motif analysis, and database hg19 was used to score motifs in gene promoter regions (500 bp upstream of the transcription start site (TSS) and 10 kb around the TSS). Finally, the AUCell algorithm was used to quantify the activity of these regulons and convert regulon activity into ON/OFF binary activity matrix with default settings. A regulon heatmap was generated using pheatmap package. We also calculated cell type specificity scores for each regulon across diverse cell types using the “calcRSS” function in the SCENIC package.

### Regulon module analysis of porcine thymocytes

To explore potential coordination patterns among regulons, we performed a regulon module analysis by the connection specificity index (CSI) method (29). Specifically, the Pearson correlation coefficient (PCC) between regulons was first calculated based on the activity scoring matrix obtained from SCENIC, and then used as an input for generating a CSI matrix according to the formula provided by Fuxman et al. (30). Secondly, regulon modules were identified based on the CSI matrix using the “ward.D” clustering method. The average score of the cell type in each module was visualized using UMAP.

### GWAS signal enrichment analysis and gene-set analysis

The pig dataset used in this study comprised 4,555 individuals with 47,257 SNPs, and a total of 30 traits were used for GWAS enrichment analysis, including 3 body shape traits, 15 reproduction traits, and 12 production traits. The summary description of phenotype data was shown in **Supplementary Table 2**. We added a 20-kb window around the gene region to include potential cis-regulatory variants. We then implemented a covariance association test (CVAT) of marker genes using the QGG package (v1.1.1) to determine the enrichment of GWAS signals in marker genes of different cell types identified based on scRNA-seq data (31). The detailed description of the method was provided at http://psoerensen.github.io/QGG/articles/gsea.html (32). In addition, we added multi-marker analysis of genome annotation (MAGMA) to further detect genetic associations between cell type-specific DEG sets and complex traits (33). Specifically, we first performed a single-locus GWAS using the MLM model in rMVP (v1.0.8) to obtain the *P*-value of each SNP, and subsequently converted the SNP-level *P*-value identified from the GWAS into a gene-level *P*-value (33, 34). We added a 20-kb window around the gene region to include potential cis-regulatory variants. Finally, a gene set association analysis was performed to test whether the genes in a gene-set are associated with the phenotype of interest. The detailed description of the method was provided at https://ctg.cncr.nl/software/magma.

## Results

### T cell subpopulations derived from thymus and lymph nodes exhibit distinct transcriptional profiles

To investigate the transcriptomic differences of T lymphocyte across different tissues, we conducted RNA-seq of a total of 7 distinct T cell subpopulations isolated from porcine thymus and lymph nodes (12). These 7 T cell subpopulations consisted of CD4-CD8-_t (CD3+CD4-CD8-), CD4+CD8+_t (CD3+CD4+CD8+), CD4_t (CD3+CD4+CD8-), CD8_t (CD3+CD4-CD8+) from porcine thymus, and CD4-CD8-_l (CD3+CD4-CD8-), CD4_l (CD3+CD4+CD8-), CD8_l (CD3+CD4-CD8+) from lymph nodes (“t” indicates thymus, and “l” denotes lymph nodes) (**Figures 1A, B**). We analyzed the expression patterns of 7 cell lineage-specific marker genes and found that the genes encoding surface receptors used for cell sorting were highly expressed in specific T cell subpopulations such as 3 CD8+ T cell subpopulations (CD8_t, CD8_l, and CD4+CD8+_t), of which *CD8A* and *CD8B* had the highest expression levels in 3 CD8+ T cell subpopulations (**Supplementary Figure 1A**). Previous research has shown that DN cells differentiate into DP cells in the thymus where DP cells in turn differentiate into CD4 or CD8 T cells through negative and positive selection (4). Subsequently, these single-positive T cells enter peripheral immune organs via the blood circulation. In this study, we performed pairwise comparisons of 7 cell subpopulations following pre-specified T cell lineage development route (**Supplementary Figure 1B**). The results showed that the largest number of DEGs were identified in the comparison of CD4_l vs. CD4_t and CD8_l vs. CD8_t, but the smallest number of DEGs were observed in CD4_t vs. CD8_t (**Figure 1C**). When comparing CD4+CD8+ and CD4-CD8- subpopulations from the thymus, we found that VDJ recombination-associated genes such as *RAG1*, *RORC*, *RORA*, and *RPA3* were upregulated in CD4+CD8+ subpopulation, but DN cell-specific marker genes including *BATF3*, *BLK*, *HES1*, and *YBX3* were downregulated in the CD4+CD8+ subpopulation (**Figure 1D** and **Supplementary Table 3**). The genes involved in cell migration and lineage commitment (such as *S1PR1*, *CH25H*, and *CRTAM*) and the genes involved in defense responses and cytotoxic functions (such as *NKG7* and *KLK7*) were unregulated in CD8 subpopulation, compared to those in CD4+CD8+ subpopulation. The genes involved in T cell activation and immune regulation such as *IL7R*, *STST3*, and *CCR7* showed higher expression levels in the CD4 subpopulation than in CD4+CD8+ subpopulation (**Figure 1D** and **Supplementary Table 3**). The comparative analysis of the transcriptional profiles of CD4-CD8-, CD4, and CD8 cell subpopulations revealed that cell activation- and effector-related gene expression levels in peripheral lymphoid tissues were increased (**Figure 1D** and **Supplementary Figure 1C**, **Supplementary Table 4**).

### Important modules associated with specific T cell subpopulations

**Figure 2:**
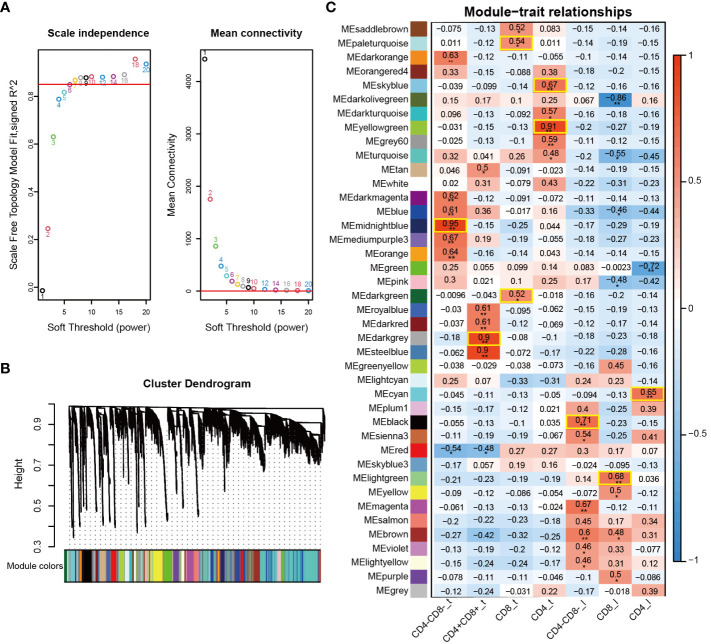
Visualization of weighted gene co-expression network of 7 T cell subpopulations. **(A)** Analysis of the scale-free fit index (left) and mean connectivity (right) for various soft-thresholding powers (β). **(B)** Clustering dendrogram of co-expression modules identified by WGCNA. **(C)** Heatmap of module-trait relationships of 7 T cell subpopulations. Each row represents a module (the same color code as in **(B)**, and each column represents a T cell subpopulation. *, *p* < 0.05; **, *p* < 0.01.

WGCNA is an effective systematic biological method for constructing gene co-expression networks, and it can be used to detect gene modules highly correlated with cell subpopulation characteristics or phenotypes (24). After excluding outlier samples and low-quality genes, a total of 13,245 genes and 19 samples were obtained and used for WGCNA analysis in this study. The “pickSoftThreshold” function determined the best soft threshold as 7 and scale-free topology fit index (R2) as 0.85 (**Figure 2A**). A total of 41 co-expressed gene modules were identified and visualized using the cluster dendrogram method, with each module labeled with a unique color (**Figure 2B**). The genes in the gray modules did not show significant co-expression patterns, and thus they were not assigned to any other modules. Subsequently, we investigated module-trait relationships for each cell subpopulation to identify key modules significantly correlated with the 7 cell subpopulations (**Figure 2C** and **Supplementary Table 5**). We found that each cell subpopulation was correlated with one or more modules. For example, the midnightblue module was highly correlated with CD4-CD8-_t, while the darkgrey module showed the highest positive correlation with CD4+CD8+_t. The paleturquoise and darkgreen modules were highly correlated with the CD8_t, and the yellowgreen and skyblue modules were highly correlated with the CD4_t. The black module exhibited the strongest correlation with the CD4-CD8-_l. Furthermore, the lightgreen module and the cyan module displayed the most correlation with CD8_l and CD4_l, respectively (**Figure 2C**). Based on these results, we selected the modules of interest for further analysis.

### Candidate hub genes regulate transcriptional heterogeneity in T cell subpopulations across tissues

**Figure 3:**
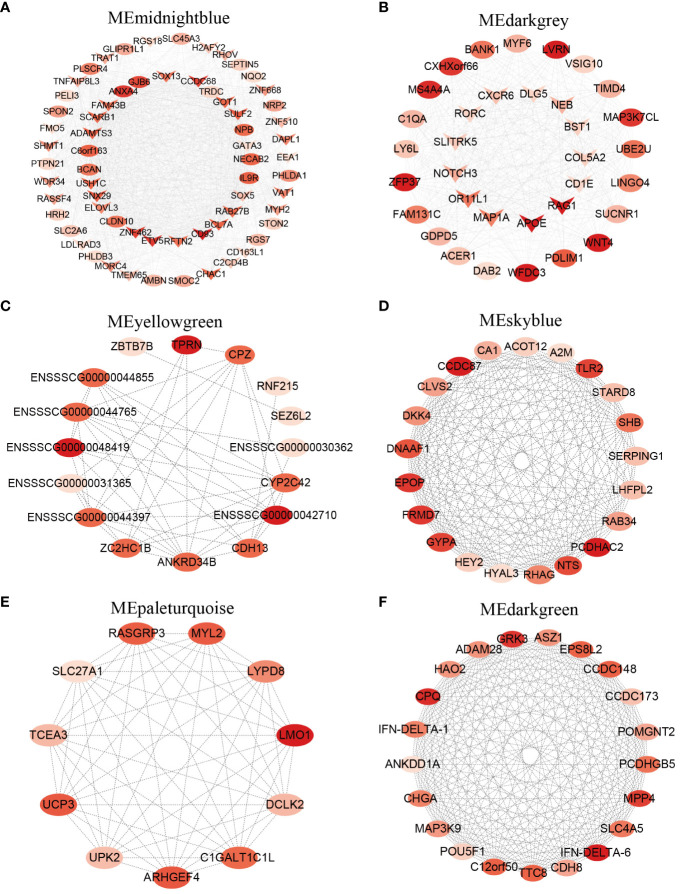
Hub genes in 4 T cell subpopulations from porcine thymus. **(A)** Network visualization of hub genes in the midnightblue module closely associated with CD4-CD8-_t. **(B)** Network visualization of hub genes in the darkgrey module closely associated with CD4+CD8+_t. **(C, D)** Network visualization of hub genes in the yellowgreen module **(C)** and the skyblue module **(D)** closely associated with CD4_t. **(E, F)** Network visualization of hub genes in the paleturquoise module **(E)** and the darkgreen module **(F)** closely associated with CD8_t. Ovals represent hub genes, “V” indicates hub DEGs overlapped with the DEGs identified by comparative transcriptome analysis. The color brightness is proportional to the maximal clique centrality (MCC) value, and the redder the color, the larger the MCC value.

By intersecting hub genes in key modules with the DEGs detected by comparative transcriptomes, we further identified core genes playing a crucial role in T cell development. The list of hub genes for each cell subpopulation was presented in **Supplementary Table 6**. The top 10 hub genes in the midnightblue module most related to CD4-CD8-_t included *CD93*, *ANXA4*, *ETV5*, *ZNF462*, *CCDC68*, *GJB6*, *SNX29*, *SULF2*, *IL9R*, and *BCL7A*, most of which were related to immune functions. In addition, three overlapping hub genes *SOX15*, *SOX3*, and *GATA3* were also present in this module, and they have been reported to regulate T cell fate commitment (**Figure 3A**) (35). In the darkgrey module most related to CD4+CD8+_t, the top 10 hub genes were *ZFP37*, *LVRN*, *APOE*, *WNT4*, *RAG1*, *WFDC3*, *MS4A4A*, *MAP3K7CL*, *CXHXorf66*, *PDLIM1*, of which *WNT4*, *APOE*, and *RAG1* genes are involved in T cell differentiation process (**Figure 3B**) (36). *ENSSSCG00000048419*, *TPRN*, *ENSSSCG00000042710*, *CPZ*, and *ZC2HC1B* were identified to be present in yellowgreen module (**Figure 3C**), and *PCDHAC2*, *CCDC87*, *EPOP*, *FRMD7*, *GYPA*, and *TLR2* were identified to be present in skyblue module, and these two modules were related to CD4_t (**Figure 3D**). *TLR2* and *HYAL3* play a role in the inflammatory response and innate immune response (37, 38). Previous studies have shown that human naive CD4 T cells express *TLR2* upon TCR stimulation, and subsequently *TLR2* functions as co-stimulatory receptor (39). In addition, *TLR2* plays a crucial role in the generation and maintenance of CD4 memory T cell (40). The top 5 hub genes in the paleturquoise module included *LMO1*, *ARHGEF4*, *UCP3*, *MYL2*, and *RASCGRP3*, and the top 5 hub genes in the darkgreen module were *IFN-DELTA-6*, *CPQ*, *GRK3*, *MPP4*, and *CCDC148*, repectively, and these two modules were associated with CD8_t (**Figures 3E, F**). *IFN-DELTA-6* and *IFN-DELTA-1* are two members of the interferon family, and they play a central role in innate and adaptive immunity with various biological effects such as antiviral and immune regulation (41). The top 10 hub genes including *CES1*, *DCHS2*, *MIA*, *GALNT8*, *TUBB4A*, *HEPACAM2*, *ZNF565*, *CES3*, *HCRT*, and *RGS22* were observed in the black module associated with CD4-CD8-_l (**Supplementary Figure 2A**). The top 10 hub genes including *DEFB124*, *C3orf49*, *FGG*, *TMEM247*, *WIPE3*, *LCA5*, *SMPDL3A*, *CNJ16*, *BEX5*, and *SLC10A8* were present in the cyan module most related to CD4_l (**Supplementary Figure 2B**). *DEFB124*, a member of the beta defense protein family involved in innate immune response, was identified as the highly connected hub gene in the cyan module (42). Hub genes *FGG*, *WIPE3*, *PP1R13L*, *PCSK5*, *NPR1* negatively regulated cell migration and cell export process. The top 10 hub genes *NWD1*, *SLFN14*, *MLIP*, *RHBG*, *PTGES3*, *TRMT5*, *MRPL18*, *FAM227A*, *SMR1*, and *TCEAL8* were found to be present in the lightgreen module most related to CD8_l (**Supplementary Figure 2C**), of which *SLFN14* and *TRMT5* were overlapping hub genes, playing an important role in mediating immune responses (43, 44).

### Bulk RNA-seq and scRNA-seq data reveal thymocyte heterogeneity in pigs

**Figure 4:**
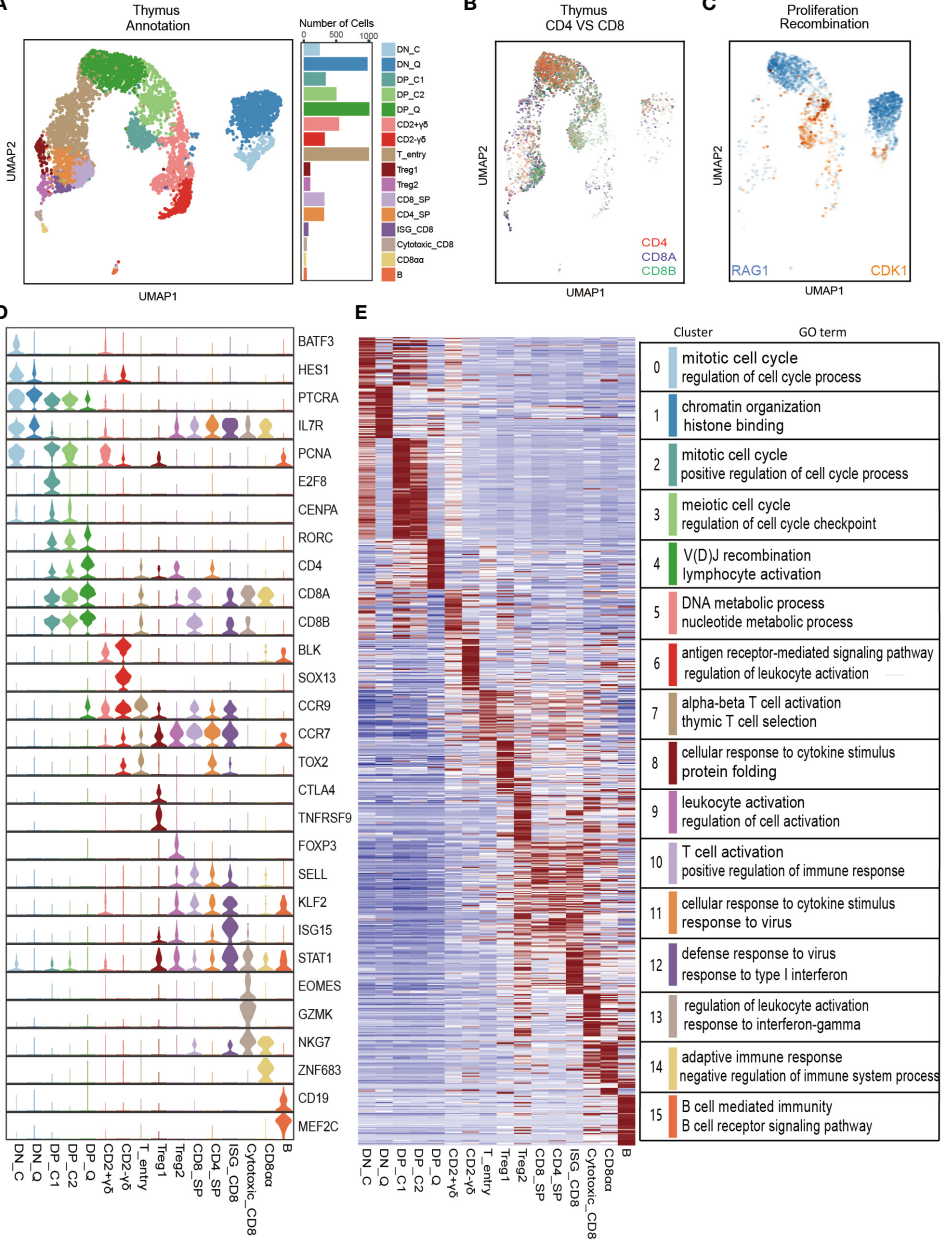
Single-cell RNA-seq analysis of porcine thymocyte populations. **(A)** UMAP analysis of 5,999 single cells from porcine thymus (left). Different colors indicate different cell types. Bar graph shows the number of cells contained in each cell type (right). **(B, C)** UMAP plot of *CD4*, *CD8A*, and *CD8B* genes **(B)** and *CDK1* cell cycle gene and *RAG1* recombination gene **(C)**. **(D)** Violin plot of classic marker genes for defining each cell type. **(E)** Heatmap of top 50 specifically expressed genes in each cell type (left) and a list of representative GO terms for each cell type (right).

Previous research on gene expression during T cell development primarily relies on bulk RNA-seq of cell populations. However, the transcriptional heterogeneity in single cells remains elusive. To reveal this heterogeneity, we downloaded the porcine thymus scRNA-seq dataset from GSE192520 and performed Seurat analysis (17). After quality control of genes and cells, cell cycle effects were eliminated, and 23 cell clusters were identified using the UMAP algorithm. Based on known specific marker gene expression, these cell clusters were manually annotated into 16 major cell types, including DN_C (*BATF3*, *HES1*, *CDK1*), DN_Q (*RAG1*, *RAG2*), DP_C1 (MXD3, *E2F8*, *CDK1*), DP_C2 (*E2F2*, *CDK1*), and DP_Q (*RAG1*, *RAG2*), CD2+γδ T cells (*CCR9*, *IKZF2*), CD2-γδ T cells (*SOX13*, *BLK*, *ETV5*), T_entry (*CCR9*, *CCR7*, *TOX2*), Treg1 (*CTLA4*, *TNFRSF9*), Treg2 (*FOXP3*, *IL2RA*, *STAT5A*), CD8_SP (*CD8A*, *CD8B*), CD4_SP (*CD4*, *LEF1*), Cytotoxic_CD8 (*GZMK*, *EOMES*, *NKG7*), ISG_CD8 (*ISG15, MX1, STAT1)*, CD8αα (*NKG7*, *ZNF683*), and B (*CD79A*, *CD19*, *MEF2C*) (**Figures 4A-D** and **Supplementary Figures 3A, B**, **Supplementary Table 7**). Correlation heatmap showed high pairwise correlation among cell types (**Supplementary Figure 3C**).

To investigate the gene expression patterns of different cell types identified by Seurat above, we extracted the top 50 marker genes (prioritized by fold change) in each cell type and drew a heatmap. As expected, the heatmap exhibited distinct signatures for each cell type (**Figure 4E**). Further, we performed GO enrichment analysis of DEGs in each cell type using the Metascape website (**Supplementary Table 8**). The genes enriched in DN_C and DP_C cell types were mainly associated with cell cycle functions, including the regulation of cell cycle process and mitotic cell cycle, and the genes in DN_P and DP_P cell types were associated with chromatin organization and VDJ recombination. GO analysis revealed that the two γδ T cell populations presented distinct biological processes. CD2-γδ-enriched genes were involved in the antigen receptor-mediated signaling pathway and regulation of leukocyte activation, while CD2+γδ-enriched genes participated in the DNA metabolic process and nucleotide metabolic process. Genes in four CD8 cell clusters (CD8_SP, ISG_CD8, Cycytoxic_CD8, and ISG_CD8) and CD4_SP were mainly involved in immune-related biological processes. The genes enriched in B cell type were primarily involved in MHC protein complex assembly and regulation of B cell activation (**Figure 4E** and **Supplementary Table 8**).

Based on annotated porcine single-cell cell types and gene sets from our bulk RNA-seq data of sorted porcine thymus cell populations, we determined the identity of porcine thymocytes by previously reported method (11). We found that some gene sets showed relatively high enrichment in their corresponding scRNA-seq clusters. Specifically, CD4-CD8-_t gene sets corresponded to DN clusters (αβ) and CD2-γδ T cluster; CD4+CD8+_t gene sets corresponded to DP cluster; and CD8_t gene sets mainly corresponded to CD8-related clusters. Interestingly, the top 5% highly enriched genes (HEGs) in CD4_t subpopulation corresponded to the B clusters (**Supplementary Figures 4A, B**). In addition, we further investigated the relationship between hub genes associated with T cell subpopulations from porcine thymus and cell types identified by Seurat. We found that most of the hub genes (such as *GATA3*, *SOX13*, *ETV5*, *ZNF462*, *RASSF4*, *SHMT1*, *CD163L1*) in the midnightblue module most related to CD4-CD8-_t were highly expressed in the DN (αβ) cell type and γδ T cell type. Hub genes (including *RORC*, *CD1E*, *RAG1*, *COL5A2*) in the darkgrey module most related to CD4+CD8+_t were specifically highly expressed in the corresponding DP cell type. Furthermore, we detected only a small number of hub genes in the modules most related to CD4_t and CD8_t subpopulations. Due to the high heterogeneity of CD4- and CD8-associated cell types identified by scRNA-Seq data, we failed to find overlap between hub genes in CD4_t and CD8_t subpopulations from bulk RNA-Seq and DEGs in cell type from scRNA-Seq. In summary, although cell subpopulations sorted by bulk RNA-Seq showed agreement with cell types identified by scRNA-Seq to some degree, bulk RNA-Seq had limitations in precisely describing transcriptional heterogeneity of cell types.

### Comparative analysis of T cell types reveals transcriptomic differences between porcine thymus and peripheral blood

The thymus serves as the primary site for T cell development and maturation, while peripheral blood transports mature T cells to participate in immune responses. Therefore, we integrated porcine thymus dataset (5,999 cells) with 8 PBMC datasets (34,220 cells) to investigate the transcriptomic differences of T cell types across tissues. Based on the expression of classic marker genes, we identified a total of 36 clusters, which were further classified to 19 major cell types, including monocytes, conventional dendritic cells (cDCs), plasmacytoid dendritic cells (pDCs), B cells, Cycling_B, antibody-secreting cells (ASC), CD2-γδ T cells, CD2+γδ T cells, DN_C, DN_Q, DP_C, DP_Q, T_entry, CD4+αβ T cells, CD8+ αβ T cells, NK cells, Cycling_NK cells, Cycling_CD8, and Erythrocytes (**Supplementary Figures 5A-C**). We found that B cells and myeloid-related cell types (including monocytes, cDCs, and pDCs) were predominantly present in peripheral blood, whereas DN and DP T cells were mainly present in porcine thymus. The γδ T cell clusters were much larger in peripheral blood than in porcine thymus. We further investigated the transcriptomic differences among the 4 T cell clusters (CD4+αβ T cells, CD8+αβ T cells, CD2+γδ T cells, and CD2-γδ T cells) in porcine thymus and peripheral blood (**Supplementary Figure 5D**). The results showed that the genes involved in CD4-positive, alpha-beta T cell proliferation and virus response (such as *IL2RA*, *FOXP3*, *TNFRSF4*) were unregulated in CD4+αβ T cells from pig thymus, while the genes involved in MHC class II protein complex binding and actin cytoskeleton regulation (such as *CD74*, *CYRIB*, *S1PR1*, *STMN1*, and *S100A10*) were unregulated in peripheral blood-derived CD4+αβ T cells (**Supplementary Table 9**). Compared with thymus-derived CD8+αβ T cells, peripheral blood-derived CD8+αβ T cells highly expressed many effect-related and cytotoxicity-related genes such as *GZMB*, *GZMM*, *NKG7*, and *GNLY*. GO enrichment analysis showed that the DEGs in peripheral blood-derived CD8+ αβ T cells were significantly enriched in such pathways as the killing of cells of another organism, leukocyte mediated immunity, positive regulation of leukocyte migration, and regulation of myeloid leukocyte mediated immunity; while the DEGs in porcine thymus-derived CD8+αβ T cells were significantly enriched in such pathways as the regulation of antigen receptor-mediated signaling pathway and negative regulation of lymphocyte mediated immunity (**Supplementary Table 9**). Surprisingly, we found that both peripheral blood-derived CD8 cells and lymph node-derived CD8 subpopulation from bulk RNA-Seq highly expressed cytotoxicity-related genes. Furthermore, the largest number of DEGs was observed in the comparison of CD2+γδ T cells in thymus vs. peripheral blood. The DEGs in peripheral blood-derived CD2+γδ T cells were mainly involved in immune-related biological processes. In addition, we observed that peripheral blood contained a high proportion of CD2-γδ T cells, and this cell type exhibited a high expression of cell migration genes *S100A6* and *SI00A5* (**Supplementary Figure 5D**). CD2-γδ T cells in the thymus highly expressed *JAML*, a gene promoting T cell proliferation and cytokine production (45). Taken together, our study reveals that genes in thymus-derived T cells are mainly related to cell proliferation, cytokine production, and early T cell development, while genes in lymph node- and peripheral blood-derived T cells are mainly related to immune responses.

### Pseudotime trajectory of porcine thymocytes is inferred

We used Slingshot to infer the developmental trajectories of porcine thymocytes by previously reported method (28). Consistent with the trajectory inferred from human thymus data, the development trajectory was as follows: porcine αβ T cells started with DN cells, gradually expressed CD4 and CD8, turned into DP cells, and subsequently DP cells differentiated from the T_entry (highly expressing *CCR9*) into CD4+ SP and CD8+ SP cells (**Supplementary Figures 6A, B**) (46, 47). Consistent with the trajectory inferred using Monocle3 based on published scRNA-Seq data, we found that independent γδ T cell lineages were diverged from the DN-DP junction (17). Notably, T cells underwent cell proliferation before each round of rearrangement, in terms of the expression patterns of genes in the quiescent phase and the proliferating phase. However, we found that the differentiation trajectories of CD4 and CD8 single positive cells inferred from porcine thymus data were not consistent with those inferred from human data. Such inconsistency might be attributed to the limited number of cells in porcine thymus.

### Key transcription factors regulating porcine thymocyte identity

**Figure 5:**
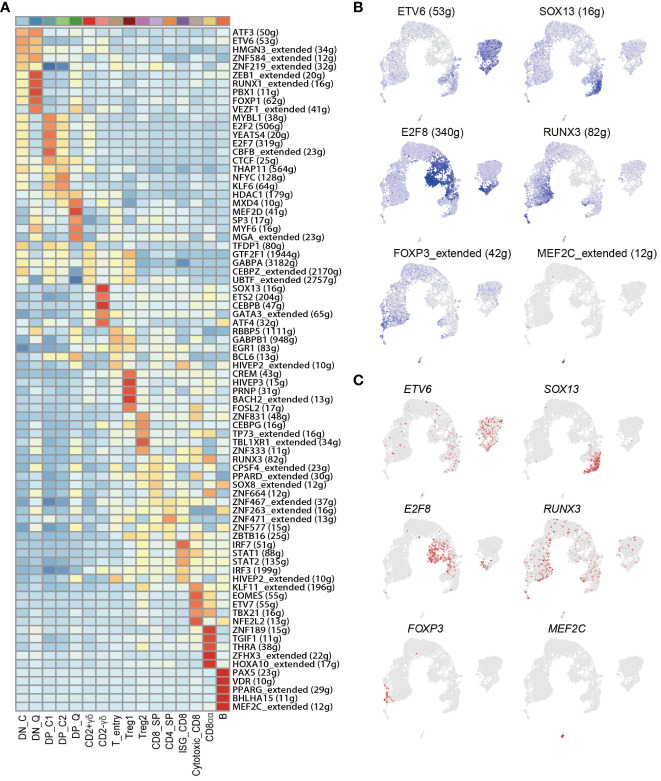
Gene regulatory networks of porcine thymocytes from scRNA-Seq data. **(A)** Top 5 specific regulons of each cell type. **(B)** UMAP plot of regulatory activities of representative TFs. **(C)** UMAP plot of the gene expression levels for representative TFs.

TFs are important regulators of gene expression and play a pivotal role in maintaining cell identity (48). Therefore, we investigated key TFs involved in T cell fate decision-making by SCENIC based on scRNA-Seq data (20). In this study, we identified 205 significant TF regulons comprising 9,588 genes, and the gene number contained in each regulon ranged from 9 to 5,608 with a median of 146. Further, we calculated the regulon specificity score (RSS) for each regulon in each cell type, and we defined the regulons with the high RSS as the critical regulons for each cell type (**Figure 5A**). We detected several universal TFs in DN (DN_C and DN_Q) and DP (DP_C1, DP_C2 and DP_Q) cell types, such as *SMARCA4*, *KLF13*, *TCF2*, and *BCL6*. Additionally, we also detected some cell type-specific TFs, such as *ZEB1*, *RUNX1*, *PBX1* in DN_Q, and *MXD4*, *MEF2D*, *MYF6*, and *MAG* in DP_Q, of which *MYF6* has been identified as a hub gene for the CD4+CD8+_t subpopulation based on bulk RNA-Seq data. We found that TFs *EOMES*, *ETV7*, *TBX21*, and *NFE2L2* were present in Cytotoxic_CD8 cell type, and TFs *ZNF189*, *TGIF1*, *THRA*, *ZFHX3*, and *HOXA10* were primarily present in ISG_CD8 cell type. TFs including *CREM*, *HIVEP3*, *PRNP*, *BACH2*, and *FOSL2* were found in Treg1 cell type, and TFs including *ZNF831*, *CEBPG*, *TP73*, *TBL1XR1*, and *ZNF333* were observed in Treg2 as specific regulons. In addition, we also found several universal TFs in CD4_SP and CD8_SP, such as *ETS1*, *IKZF1*, *KLF2*, *ELK3*, and *NFKB1*. The classical TFs, *GATA3* and *SOX13*, were found to be specifically expressed in CD2-γδ T cells, and they were identified as hub genes of CD4-CD8-_t subpopulation based on bulk RNA-Seq data (49, 50). B cell was a well-characterized cell type, and *PAX5*, *MEF2C*, *BHLHAI5*, *TCF4*, and *IRF4*, were identified as the B cell-specific regulons in this study (**Figure 5A** and **Supplementary Figure 7A**, **Supplementary Table 10**). Notably, all these 5 TFs have been reported as core regulators of B cell identify maintenance (51). As UMAP plot shown, regulatory activities of representative TFs were consistent with their gene expression levels (**Figures 5B, C** and **Supplementary Figures 7B, C**).

### Module analysis unveils coordinated transcription factor expression patterns during porcine thymus T cell differentiation process

**Figure 6:**
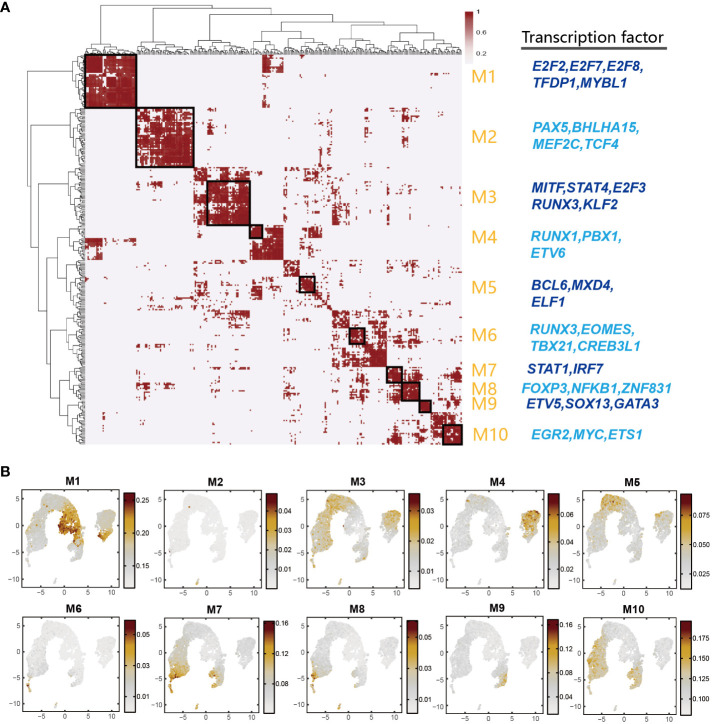
Identification of combined regulon modules based on scRNA-Seq data. **(A)** Identification of regulon modules based on the connection specificity index (CSI) matrix in porcine thymus. The representative TFs were presented in right panel. **(B)** UMAP plot of average activity of each module.

Multiple TFs often synergistically regulate gene expressions. To investigate the combination patterns of the above-mentioned TFs identified by SCENIC, we performed a module analysis by CSI method (30). Through unsupervised hierarchical clustering, 205 TF regulons were combined into 10 major modules (M1-M10) (**Figures 6A, B** and **Supplementary Table 11**). We calculated the average activity scores of each module and mapped them onto the UMAP plots. The results showed that each module occupied distinct regions, exhibiting complementary patterns among distinct regions (**Figure 6B**). We found that most of the specific regulators in DP_C1, DP_C2 and DN_C cell types were clustered into M1 such as *E2F7*, *E2F3*, *ETV5*, *TFDP1*, *CTCF*, and these regulators have been reported to be involved in cell proliferation. M2 was primarily associated with B cells, and many B cell-related regulators such as *PAX5*, *BHLHA15*, *MEF2C*, *TCF4*, and *IRF4* were found in this module. M3 and M10 modules were a mixture of several cell types and these two modules presented complementary characteristics. M4 contained regulators *FOXP1* and *ETV6*, which were important regulators for the DN_Q cell type. M5 contained regulators specifically activated in DP_Q cell types, including *BCL6*, *MXD4*, and *ELF1*. Similar regulator clustering patterns were also observed in other cell types such as Cytotoxic_CD8 cells (M6), ISG_CD8 cells (M8), and CD2-γδ cells (M9). M10 contained a mixture of cell types other than DN and DP cell types (**Figure 6A**). Collectively, these results suggested that cell types with similar functions might have similar TF activation patterns, and that TFs synergistically drove the expression of their respective target genes, thus regulating the overall T cell differentiation process.

### GWAS signal enrichment analysis reveals role of immune cell types in regulating complex traits of pigs

**Figure 7:**
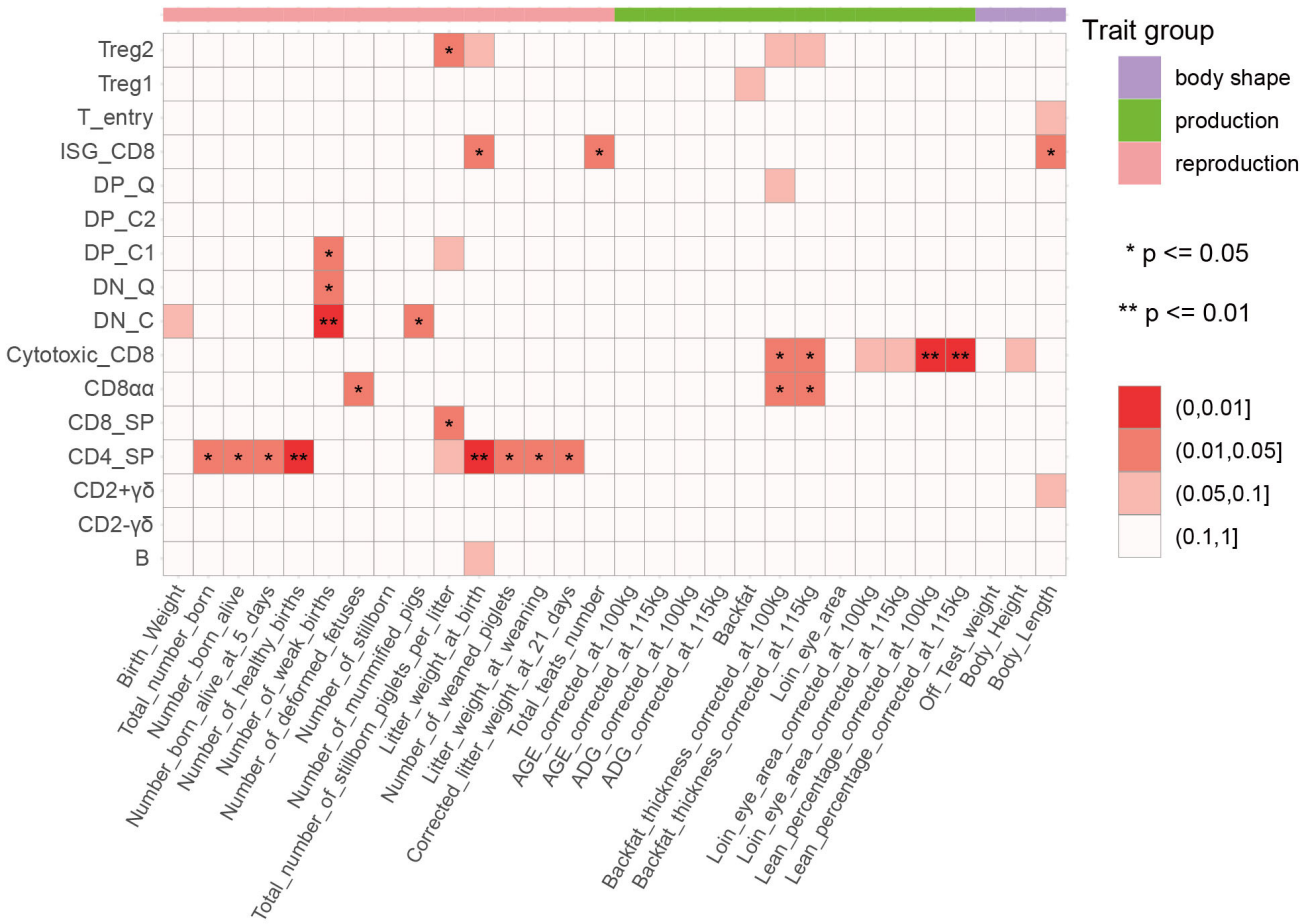
Association between 30 complex traits and 16 cell types. The color indicates the enrichment degree, which was calculated by a CVAT-based GWAS signal enrichment analysis of differentially expressed genes (DEGs) among cell types. *, *p* ≤ 0.05; **, *p* ≤ 0.01.

Complex traits are mostly controlled by multiple genes, and recent research has indicated the differences in the impacts of different tissues or cell types on complex traits (52). To determine the relationship between thymic development-related cell types identified by scRNA-Seq data and complex traits in pigs, we performed a GWAS signal enrichment analysis of DEGs in each cell type using QGG (**Supplementary Tables 2**, **7**, **12**). The results showed that DEGs in 3 unconventional CD8+ T cell types (ISG-CD8, Cytotoxic_CD8, and CD8αα) were significantly associated with production and body shape traits (**Figure 7**). To validate this result, we additionally used MAGMA, a gene and gene set analysis of GWAS genotype data, to detect associations between cell type-specific gene sets and complex traits. Gene set analysis using MAGMA confirmed that DEGs in thymus development-related cell types, especially Cytotoxic_CD8, and CD8αα, were significantly associated with production traits such as backfat thickness, loin eye area, lean percentage corrected to 100kg and 115kg (**Supplementary Figure 8A**). ISG-CD8 T cells have been reported to mediate the antiviral activity of interferon (IFN)-α and type I IFN, and type I IFN is involved in inducing interferon-stimulated genes during the late stages of human and mouse thymocyte development (53–55). Cytotoxic CD8 and CD8αα T cells highly express T cell memory marker genes (*CD44*, *CXCR3*, and *CCL5*) and NK cell marker genes (*NKG7* and *KLRK1*) (17). The three types of T cells can rapidly initiate immune responses to maintain body health in the case of re-invasion by viruses or bacteria during the growth of pigs. DEGs in multiple cell types were significantly associated with reproductive traits, indicating the important role of immune cells in facilitating embryo implantation, promoting placenta formation, and supporting embryonic development (**Figure 7**) (56–58). Furthermore, the results of QGG and MAGMA jointly showed that the DEGs in CD4_SP were significantly associated with litter weight at weaning and corrected litter weight at 21-day (**Figure 7**, **Supplementary Figure 8A**). Additionally, we performed association analysis between peripheral blood-derived cell types and complex traits using the same method. The results of both QGG and MAGMA showed that the DEGs in blood-derived CD4 and CD8 cell types were significantly associated with multiple production traits and body shape traits (**Supplementary Figures 8B, C**). We also found that DEGs in blood-derived NK cells and B cells were significantly associated with reproductive traits (**Supplementary Figures 8B, C**).

## Discussion

In this study, we first compared distinct T cell subpopulations isolated from porcine thymus and lymph nodes using bulk RNA-seq (**Figure 1** and **Supplementary Figure 1**). Our finding revealed that the transcriptome differences depended more on the tissue origin than on T cell phenotype. Lymph node T cells had a larger number of DEGs than thymus T cells, indicating greater transcriptional diversity of lymph node T cells. Moreover, we observed distinct differences in CD4 and CD8 T cell transcriptome profiles between lymph nodes and the thymus, potentially suggesting the differentiation and diversification of thymus-derived naive T cells upon encountering their cognate antigen in the peripheral tissues (59). Our functional enrichment analysis further demonstrated that 7 T cell subpopulations exhibited specific and different functions among different tissues (**Supplementary Figure 1C**). For instance, the genes in thymus-derived T cells were primarily associated with the cell cycle, cell proliferation, and early T cell development, whereas the genes in lymph node-derived T cells were mainly related to immune responses in peripheral blood.

Transcriptome-based differential gene expression analysis allows the identification of DEGs of interest between groups, but the genes in a module often exhibit co-expression relationships. WGCNA is a systematic biology approach that modularizes large datasets based on similar gene expression patterns to obtain co-expression modules with great biological significance, thereby facilitating the identification of hub genes associated with specific phenotype (60). In this study, a total of 41 co-expression modules were generated based on WGCNA, from which we identified one or two key modules associated with each specific cell subpopulation (**Figure 2**). For example, the midnightblue module exhibited a positive correlation with the CD4-CD8-_t subpopulation, whereas the darkgrey module displayed the highest positive correlation with the CD4+CD8+_t subpopulation. These results suggested possible differences in the composition of gene co-expression network at different T cell differentiation stages. Based on the correlation between genes and cell subpopulation, we screened a large number of hub genes from specific cell subpopulations associated with the key modules, and these hub genes potentially played pivotal functions at different T cell development stages (**Figure 3**). For instance, hub genes *SOX15*, *SOX3*, *GATA3*, *ETV5*, and *CD163L1* identified from CD4-CD8-_t were highly expressed in the corresponding cell types DN and CD2-γδ T cells. Hub genes *RORC*, *CD1E*, *RAG1* and *COL5A2* identified from CD4+CD8+_t were highly expressed in the corresponding DP cells from scRNA-seq data. These hub genes have been reported to be involved in regulating T cell fate commitment and differentiation processes (35, 36). Additionally, the classical TFs *GATA3*, *SOX13*, and *MYF6* were identified based on bulk RNA-Seq and single-cell data, further highlighting their important roles in thymocyte development.

Our analysis of public porcine thymus scRNA-seq data revealed cell heterogeneity during T cell development (**Figure 4** and **Supplementary Figure 3**). Based on the expression of proliferation-related genes and cell type-specific marker genes reported in literature, we identified a total of 16 cell types, which was consistent with previous studies (17). We further investigated the biological function of 16 cell types using Metascape (**Figure 4E**). As expected, the results of the GO enrichment analysis confirmed the unique identities of these cell types. We also conducted cross-tissue comparisons of cell types based on scRNA-seq data of porcine thymus and PBMC (**Supplementary Figure 5**). Our analysis revealed that DN cells were thymus-specific, while myeloid-related cell types were predominantly present in blood tissue. One previous study has shown that other immune cells were also present in the human thymus, including B cells, NK cells, macrophages, monocytes, and DCs (47). Given the limited current porcine thymus data, the inclusion of more thymus sample data will facilitate future comprehensive cross-tissue study. Furthermore, we investigated the genetic relationships between porcine thymus and peripheral blood cell types and complex traits (**Figure 7** and **Supplementary Figure 8**). We found that GWAS signals of multiple cell types, especially thymus-derived CD8 cell types (including CD8_SP, ISG-CD8, Cytotoxic_CD8, and CD8αα) and peripheral blood-derived CD4_SP and CD8_SP, were significantly associated with production and body shape traits. These results were consistent with previous research findings, suggesting that the immune system was involved in the growth processes of pigs (61). In addition, we also observed that DEGs in multiple cell types were significantly associated with reproductive traits, indicating the important role of immune cells in facilitating embryo implantation and pregnancy establishment (57, 58). Notably, DEGs in blood-derived NK cells and B cells were significantly associated with reproductive traits (**Supplementary Figures 8B, C**). As the most abundant leukocyte type in the decidua, NK cell deficiency impaired spiral arterial remodeling during pregnancy and reduced trophoblast invasion (62–64). B cells provided immune protection for mothers and newborns by producing antibodies during pregnancy and lactation (65, 66). In summary, our results emphasized the critical role of immune-related cell types in porcine growth and reproduction, contributing to a better understanding of the genetic and biological basis of these complex traits.

To investigate the consistency in gene expression profiles between the scRNA-seq data and the bulk RNA-seq data, we next extracted the top 5%, 10%, 15%, 20%, 25%, 30%, and enriched gene sets from bulk RNA-seq populations based on log_2_FC value, followed by gene set enrichment analysis of porcine scRNA-seq data using AUCell (v1.10.0) (11) (see Methods section). We found that gene sets in CD4+CD8+_t exhibited high relative enrichment in the expected corresponding scRNA-seq cell clusters (DP_C1, DP_C2, DP_Q) (**Supplementary Figure 4**). Additionally, we noticed that gene sets in CD8_t were enriched in multiple different CD8-related clusters, such as CD8_SP, ISG_CD8, Cytotoxic_CD8, and CD8αα cell clusters (**Supplementary Figure 4**). This phenomenon might be because these CD8 T cell clusters (CD8_SP, ISG_CD8, Cytotoxic_CD8, and CD8αα) shared many same gene, thus resulting in less cell type-enriched or cell type-specific genes detected, or because the lack of specific antibodies led to difficulties in distinguishing different cell types during the sorting process. We also observed that CD4-CD8-_t gene sets were highly enriched in DN (αβ) and CD2-γδ T cell types. The possible reason might be that the lack of specific antibodies targeting γδ T cells in the cell sorting strategy prevented CD2-γδ T cells from being separated from αβ cell populations. Notably, the top 5%-10% of CD4_SP HEGs exhibited relatively high enrichment in B cells (**Supplementary Figure 4**). B cells provide additional and indispensable antigen-presenting capacity to facilitate clonal expansion, and differentiation of CD4 T cells, as reported (67).

The Slingshot analysis in this study and the Monocle3 analysis in previous study jointly indicated that αβ T cell development in the porcine thymus closely resemble that in human thymus. Namely, the DN cells differentiate into the DP cells, and the latter further differentiates into CD4+CD8- SP or CD4-CD8+ SP T cells after negative and positive selection (**Supplementary Figure 6**) (3, 47). CD2+γδ T cells differentiate from DN thymocytes into CD2- γδ T cells. In addition, we constructed a gene regulatory network of porcine thymocytes using SCENIC. We identified multiple cell type-specific TFs and several universal TFs shared by some cell types, some of which were consistent with those reported in previous study of human thymocyte types (**Figure 5**, **Supplementary Figure 7**) (47). Given that these TFs showed distinct but complementary expression patterns across different cell clusters, we performed a module analysis and revealed enrichment status of the TF regulons in modules (**Figure 6**). For instance, key regulons responsible for the cell cycle and cell differentiation including *E2F2*, *E2F7*, and *TFDP2*, were enriched in M1 (47). B cell differentiation-related TFs such as *IRF8*, *MEF2C*, *PAX5*, and *SPI1* were enriched in M2 (51). Additionally, some well-reported TFs (*SOX13*, *GATA3*, *MAF*, and *ETV5*) involved in CD2-γδ T differentiation were found to be enriched in M9 (35, 68). Taken together, the above results suggested that cell types with similar functions might have similar TF activation patterns, and that coordinated TF expression governed the entire process of T cell differentiation.

However, our study also has some limitations. First, we analyzed porcine thymus T cell developmental regulatory programs, such as key TFs, but our findings lacked experiment validation. Moreover, only bulk RNA-seq data of lymph node tissue was analyzed in this study, and future work is suggested to include scRNA-seq data across porcine tissues and ages in the exploration of T cell lineage decisions so as to better understand the role of T lymphocytes in adaptive immune systems in mammals.

In summary, we first investigated the transcriptome profile differences among different T cell populations in the porcine thymus and peripheral lymph nodes, and identified several hub genes in T cell subpopulations. Based on single-cell datasets, we established gene regulatory networks of porcine thymocytes and identified key TFs driving thymocyte differentiation. The integration of GWAS with single-cell transcriptome analysis provides novel insights into the genetic and biological basis of complex traits in pigs.

## Data availability statement

The datasets presented in this study can be found in online repositories. The names of the repository/repositories and accession number(s) can be found below: https://www.ncbi.nlm.nih.gov/, GSE247127.

## Ethics statement

The animal study was approved by The Institutional Animal Care and Use Committee of Huazhong Agricultural University. The study was conducted in accordance with the local legislation and institutional requirements.

## Author contributions

PH: Conceptualization, Data curation, Formal analysis, Investigation, Methodology, Visualization, Writing – original draft. WZ: Data curation, Investigation, Resources, Writing – review & editing. DW: Data curation, Investigation, Resources, Writing – review & editing. YW: Data curation, Investigation, Resources, Writing – review & editing. XL: Conceptualization, Funding acquisition, Project administration, Supervision, Writing – review & editing. SZ: Conceptualization, Funding acquisition, Project administration, Supervision, Writing – review & editing. MZ: Conceptualization, Funding acquisition, Project administration, Supervision, Writing – review & editing.

## Supplementary material

Supplementary Table 1 Updated complete list of gene names.

Supplementary Table 2 Summary description of the phenotype data used for GWAS signature enrichment analysis.

Supplementary Table 3 Differentially expressed genes for pairwise comparisons of indicated subpopulations.

Supplementary Table 4 Significantly upregulated biological processes in pairwise comparisons of indicated subpopulations.

Supplementary Table 5 Module results associated with 7 T cell subpopulations detected by WGCNA.

Supplementary Table 6 Hub genes of 7 T cell subpopulations.

Supplementary Table 7 Differentially expressed genes of porcine thymus cell types identified by Seurat.

Supplementary Table 8 Gene Ontology enrichment analysis for 16 cell types in porcine thymus.

Supplementary Table 9 Differentially expressed genes (DEGs) and biological processes in which DEGs were enriched in T cells from porcine thymus and peripheral blood.

Supplementary Table 10 Cell type specific scores for each regulon in each identified cell type.

Supplementary Table 11 The results of regulons module analysis.

Supplementary Table 12 The significant GWAS signal enrichment of 30 complex traits across cell types.
