# Supplementary material for: Comparative transcriptome analysis of T lymphocyte subpopulations and identification of critical regulators defining porcine thymocyte identity
Source: Front Immunol. 2024 Feb 7;15:1339787. doi: 10.3389/fimmu.2024.1339787 (PMC10879363; doi:10.3389/fimmu.2024.1339787)
Supplement: Supplementary file 1 [file DataSheet_1.zip › Supplemental Figures.docx]

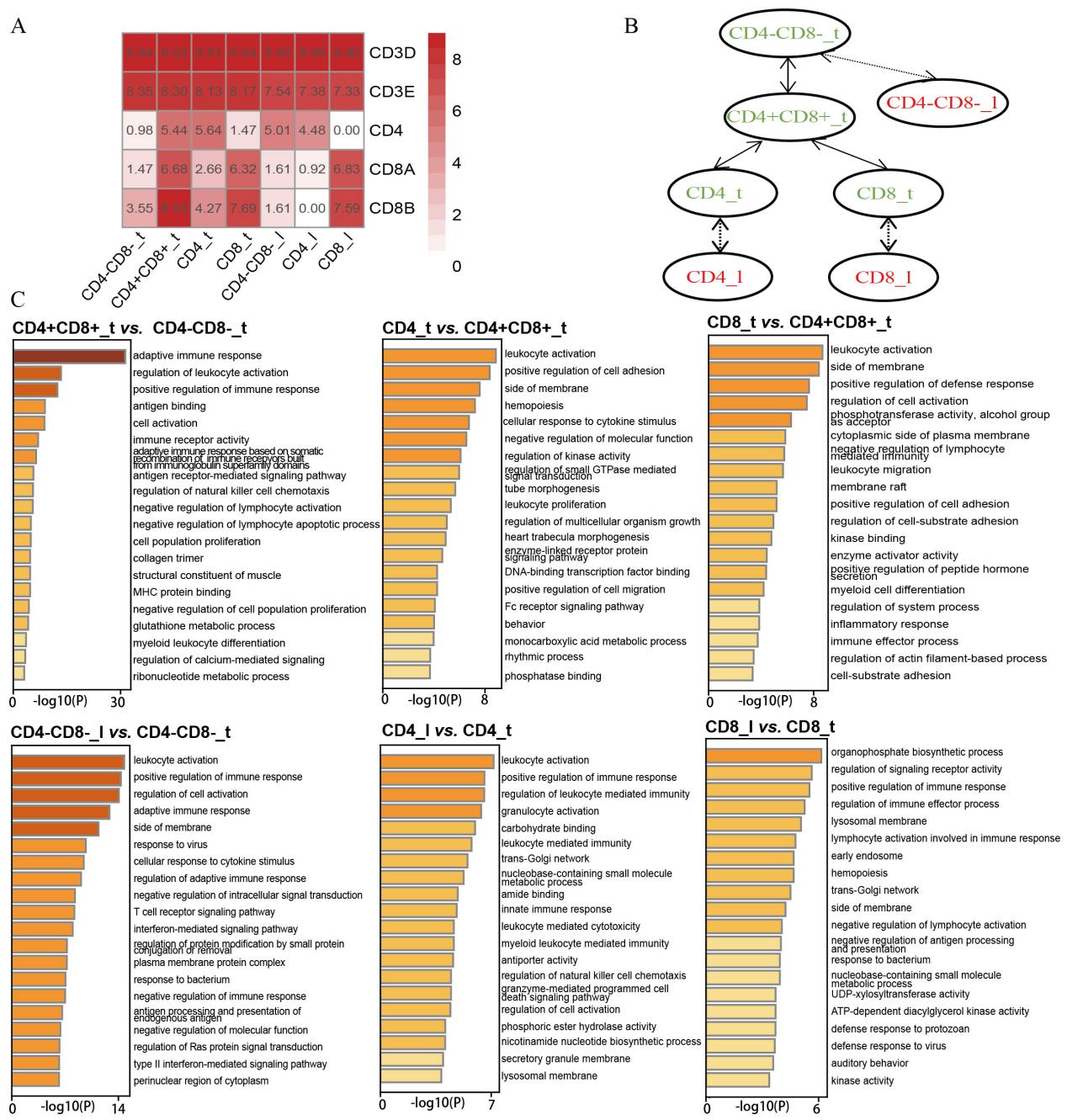


**Supplementary Figure 1.** Bulk RNA‑Seq analysis of T cell subpopulations derived from the thymus and lymph nodes. **(A)** Heatmap of expression levels of marker genes used for sorting the 7 T cell populations. The gene expression values were log_2_ values after normalization. **(B)** Schematic diagram of T cell subpopulation comparison. Solid arrows indicate pairwise comparisons of cell subpopulations from thymus, and dashed lines indicate pairwise comparisons of cell subpopulations between the thymus and lymph nodes. **(C)** Bars of the top top 20 representative GO terms in which up-regulated differentially expressed genes (DEGs) were significantly enriched in pairwise comparisons of specified cell subpopulations.


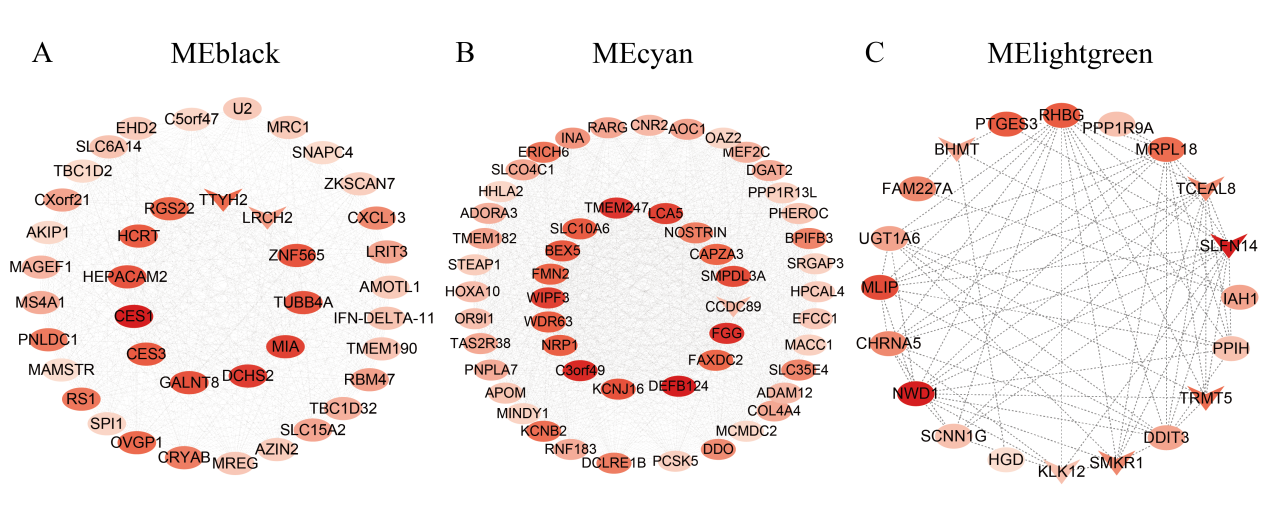


**Supplementary Figure 2.** Hub genes in 3 T cell subpopulations from porcine lymph nodes. **(A)** Network visualization of hub genes in the black module closely associated with CD4-CD8-_l. **(B)** Network visualization of hub genes in the cyan module closely associated with CD4_l. **(C)** Network visualization of hub genes in the lightgreen module closely associated with CD8_l. Ovals represent hub genes, “V” indicates hub DEGs overlapped with the DEGs identified by comparative transcriptome analysis. The color brightness is proportional to the maximal clique centrality (MCC) value, and the redder the color, the larger the MCC value.


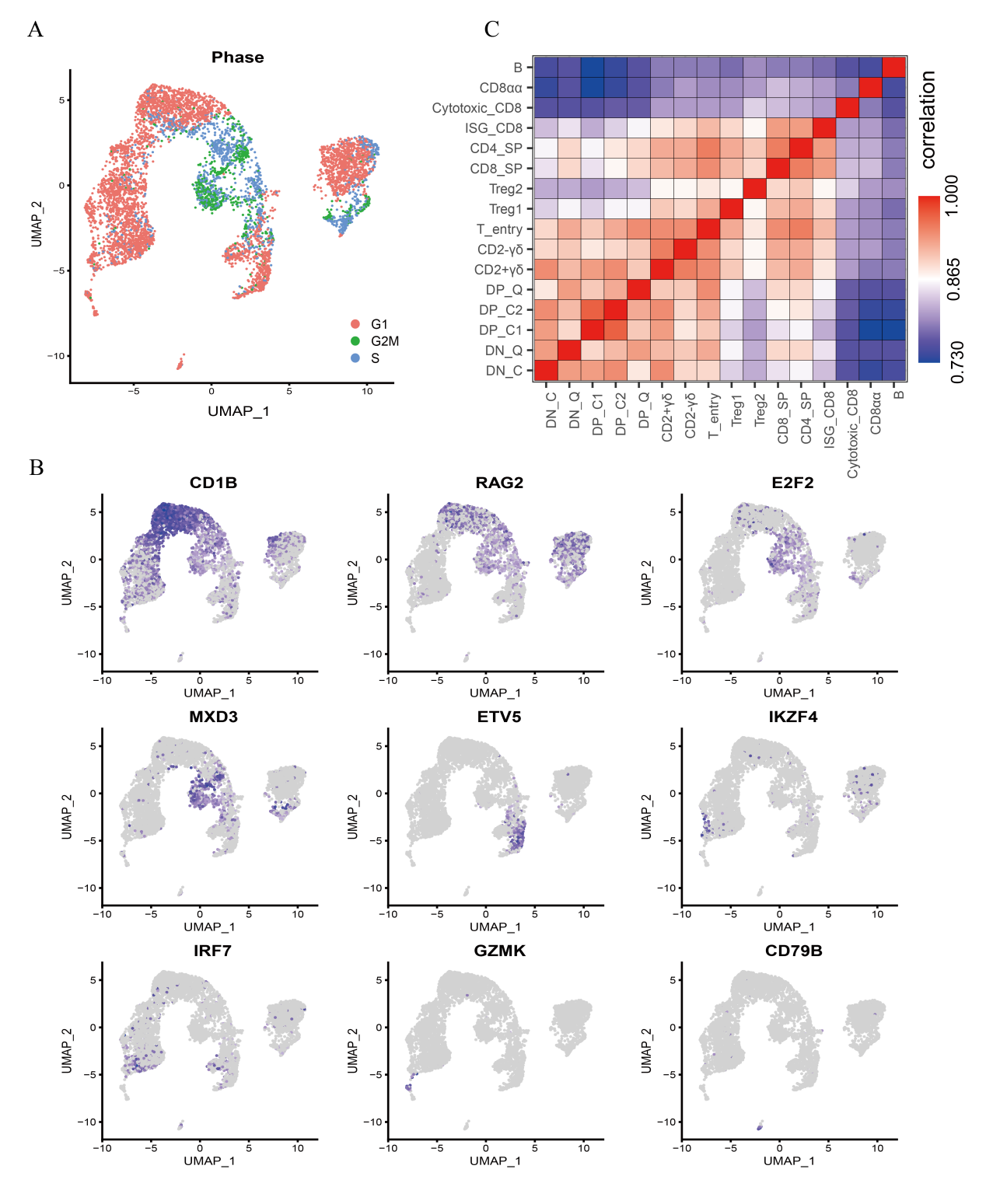


**Supplementary Figure 3.** Single-cell transcriptomic analysis of porcine thymocytes. **(A)** UMAP plot of porcine thymocytes. Different colors indicate different cell-cycle phases. **(B)** UMAP plots of marker genes for defining cell types. **(C)** Pearson correlation analysis of cell types. Colors represent the strength of correlations between different cell types.


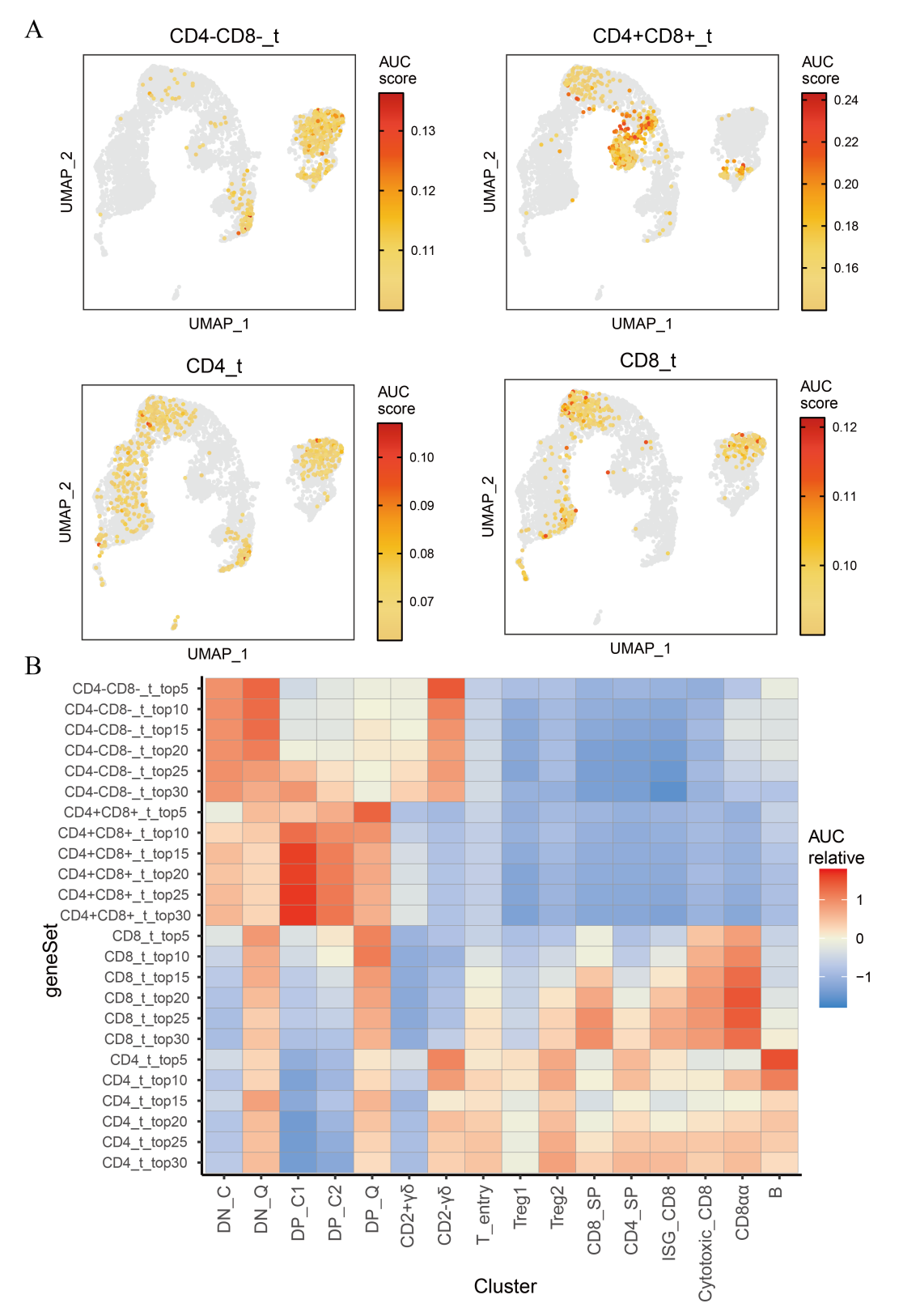


**Supplementary Figure 4.** Enrichment scores of high enrichment genes (HEGs) from the porcine bulk RNA-seq data in corresponding scRNA-seq cell clusters. **(A)** Gene set enrichment scores of each cell calculated by AUCell analysis of the top 20% HEG set (based on log_2_FC value) in the porcine bulk RNA-seq sorted cell populations. These enrichment scores were mapped onto the cells of the porcine scRNA-seq data in a UMAP plot. Each dot represents a cell, and its color is proportional to the AUC score calculated for this cell. **(B)** Relative average gene set enrichment scores of scRNA-seq clusters calculated by AUCell analysis of the top 5%, 10%, 15%, 20%, 25%, 30% HEG sets (based on log_2_FC values) in porcine RNA-seq data.


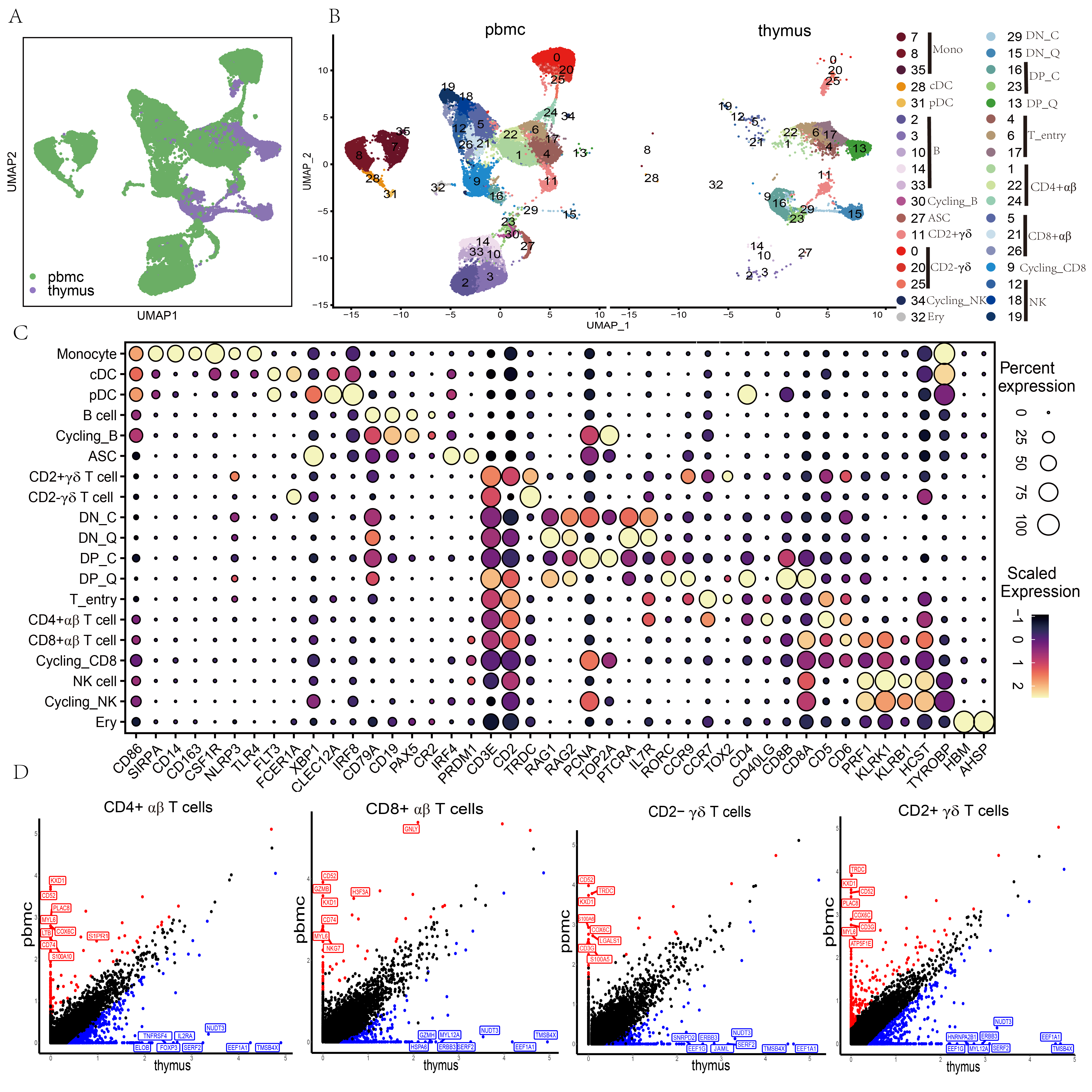


**Supplementary Figure 5.** Integrative analysis of porcine thymocytes and peripheral blood monocytes. (**A-B**) UMAP map of integrated analysis of porcine thymocytes and peripheral blood monocytes, colored by sample origin (**A**) and colored by cell type (**B**). (**C**) Dot plot of the expression of cell type-specific marker genes. Dot brightness and size represent the scaled expression of each marker gene and the proportion of cells expressing each marker gene, respectively. (**D**) Scatter plots of average expression of genes in 4 T cell types with thymocytes as X-axis and peripheral blood mononuclear cells as Y-axis (Wilcoxon rank-sum test). Red dots represent genes that were upregulated in peripheral blood mononuclear cells (Y-axis), and blue dots represent genes that were upregulated in thymocytes (X-axis). DEGs were identified with the thresholds of adjusted *P* < 0.01 and |log_2_FC| > 0.58.


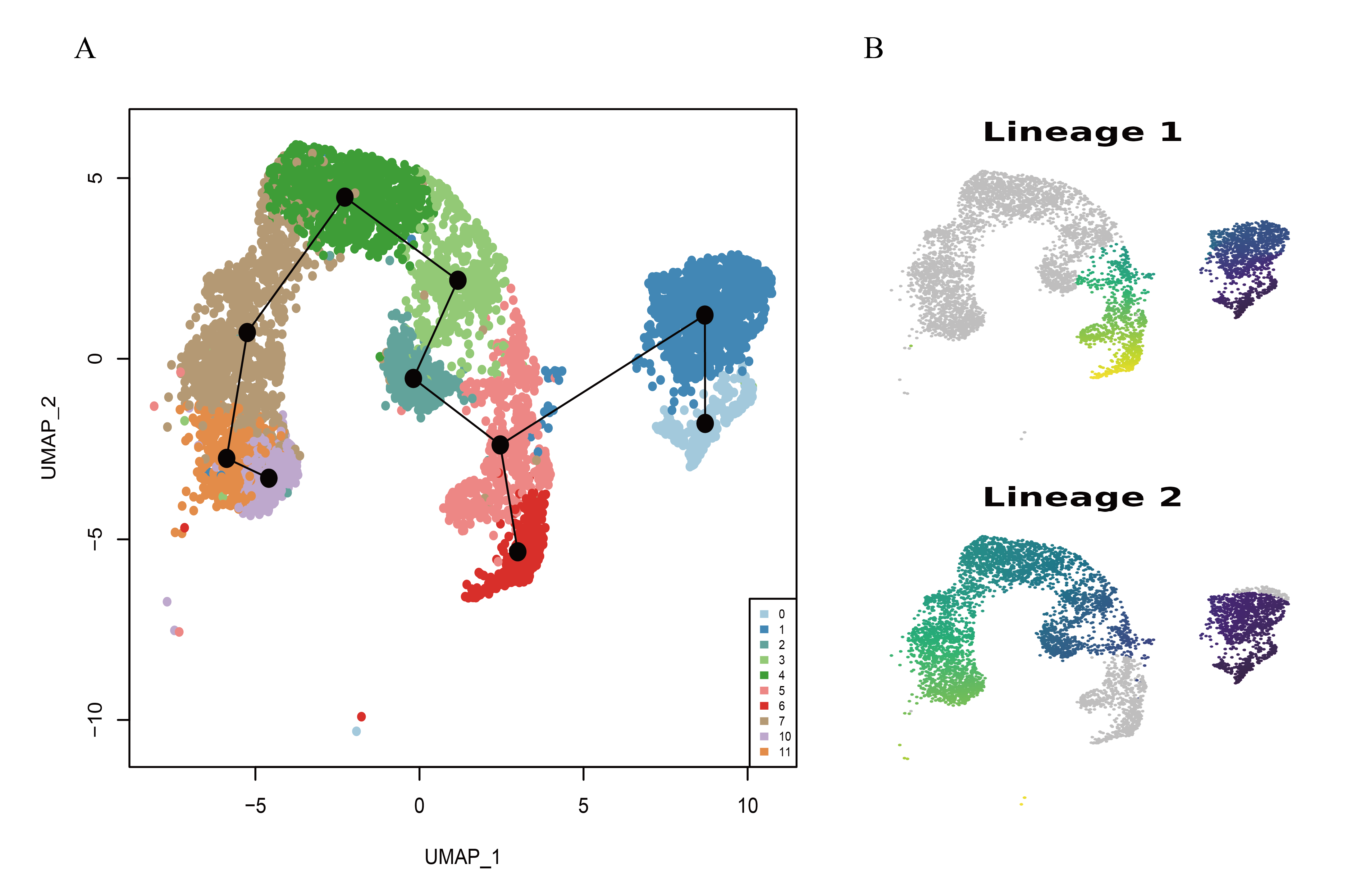


**Supplementary Figure 6.** Developmental trajectory of porcine thymocytes. (**A-B**) Pig thymocyte differentiation trajectory inferred by Slingshot according to cluster information (**A**) and pseudo time (**B**).


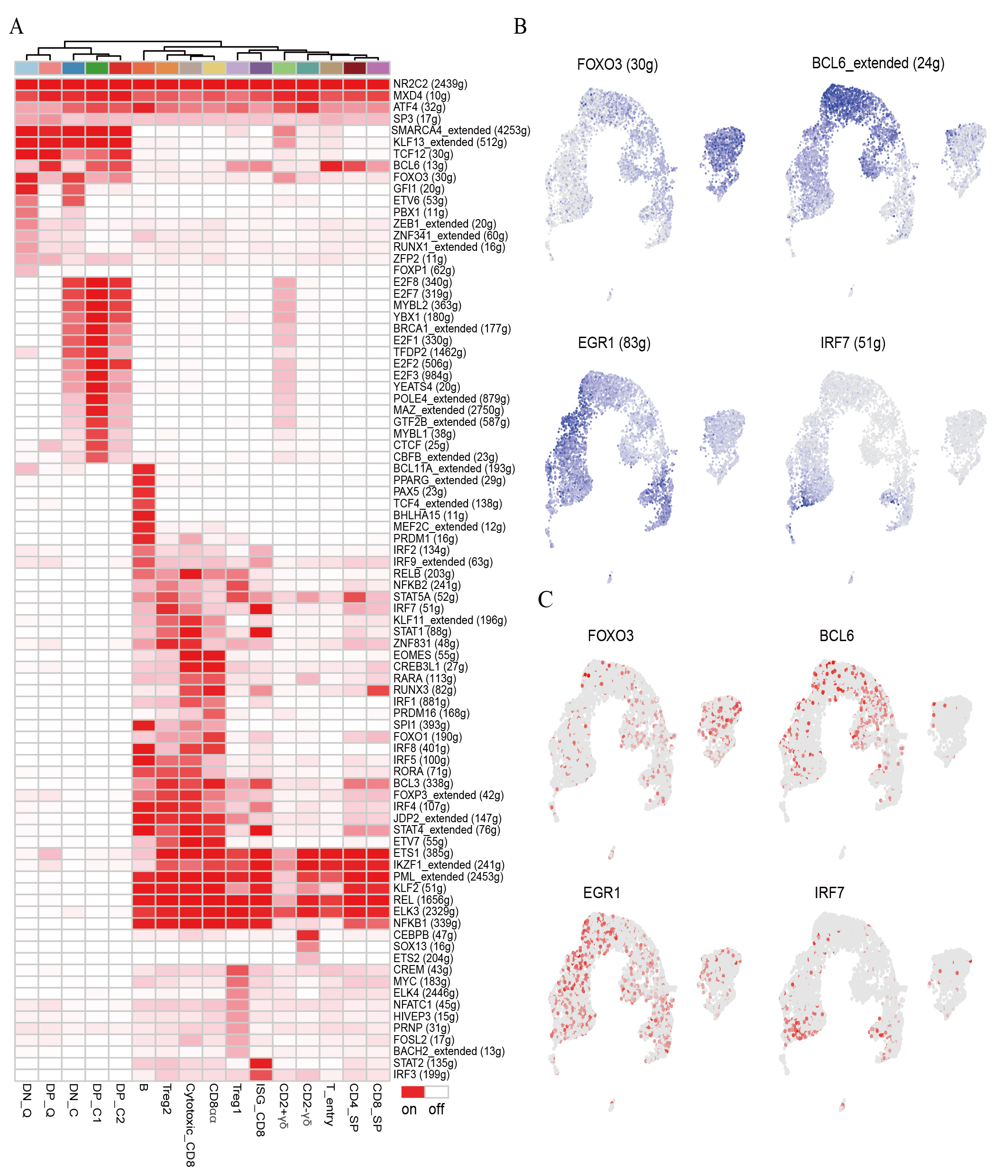


**Supplementary Figure 7.** Gene regulatory networks of porcine thymocytes. **(A)** SCENIC binary regulon activity heatmap of porcine thymocytes. **(B)** UMAP plot of regulatory activities of representative TFs. **(C)** UMAP plot of gene expression levels for representative TFs.

**
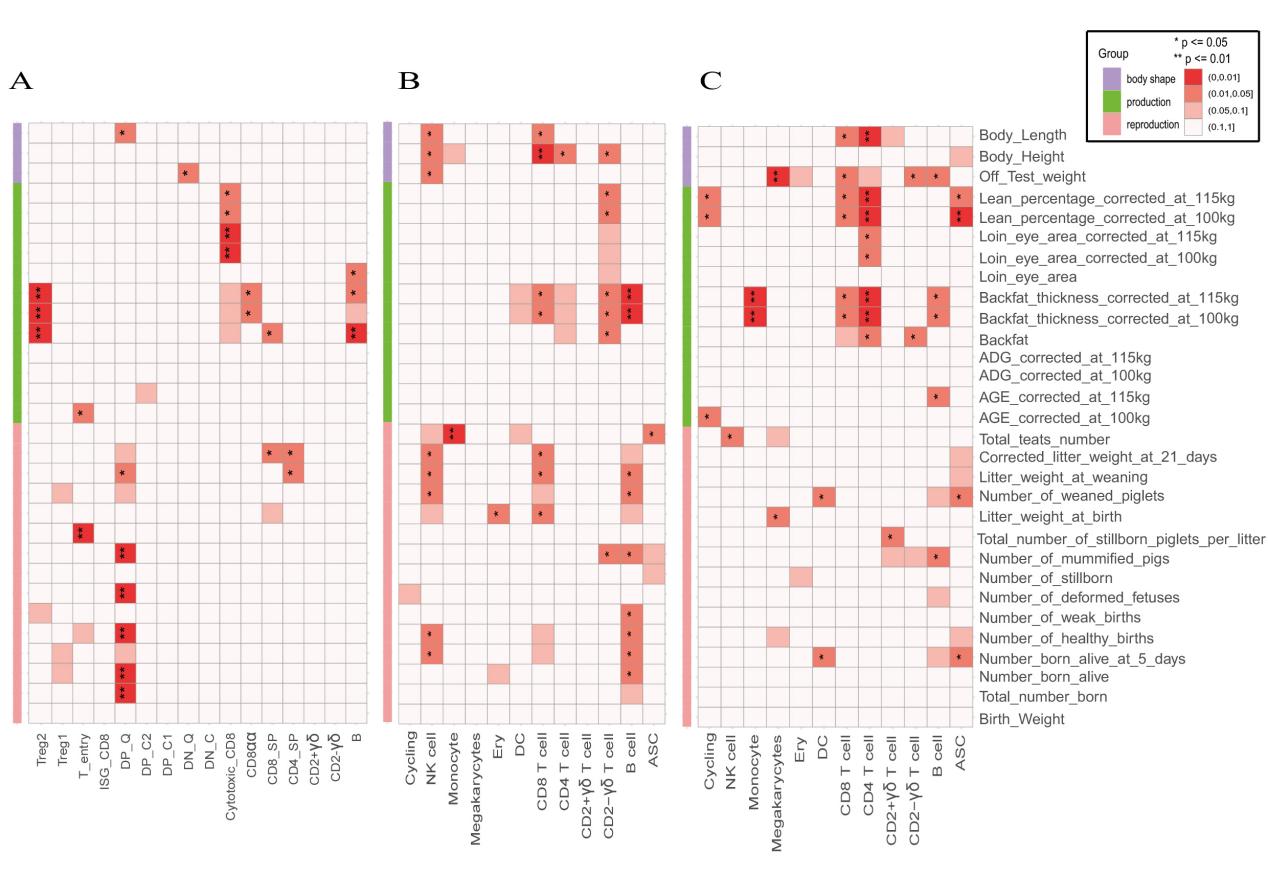
**

**Supplementary Figure 8.** Associations between 30 complex traits and thymus-derived and peripheral blood-derived cell types. (**A**) Gene set association analysis of thymus-derived cell types-specific differentially expressed genes (DEGs) and complex traits based on MAGMA. (B) GWAS signal enrichment analysis of peripheral blood-derived cell types-specific DEGs and complex traits based on QGG. The color indicates the enrichment degree. (C) Gene set association analysis of peripheral blood-derived cell types-specific DEGs and complex traits based on MAGMA. *, *p* ≤ 0.05; **, *p* ≤ 0.01.
