# Supplementary figures and images for: Comparative transcriptome analysis of T lymphocyte subpopulations and identification of critical regulators defining porcine thymocyte identity
Source: Front Immunol. 2024 Feb 7;15:1339787. doi: 10.3389/fimmu.2024.1339787 (PMC10879363; doi:10.3389/fimmu.2024.1339787)

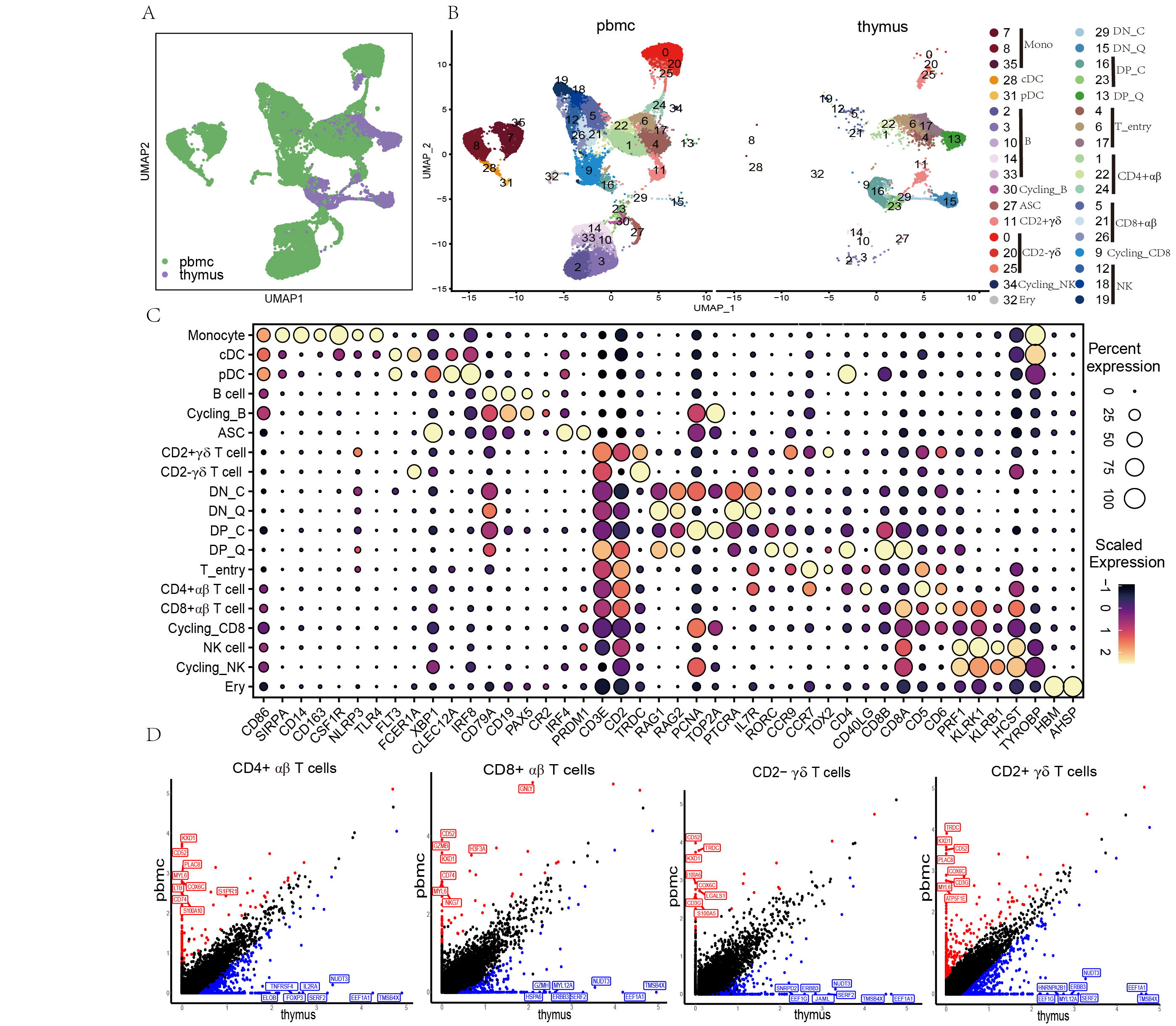

Supplement: Supplementary file 1 [file DataSheet_1.zip › Supplemental Figures/Supplementary Figure5.jpg]

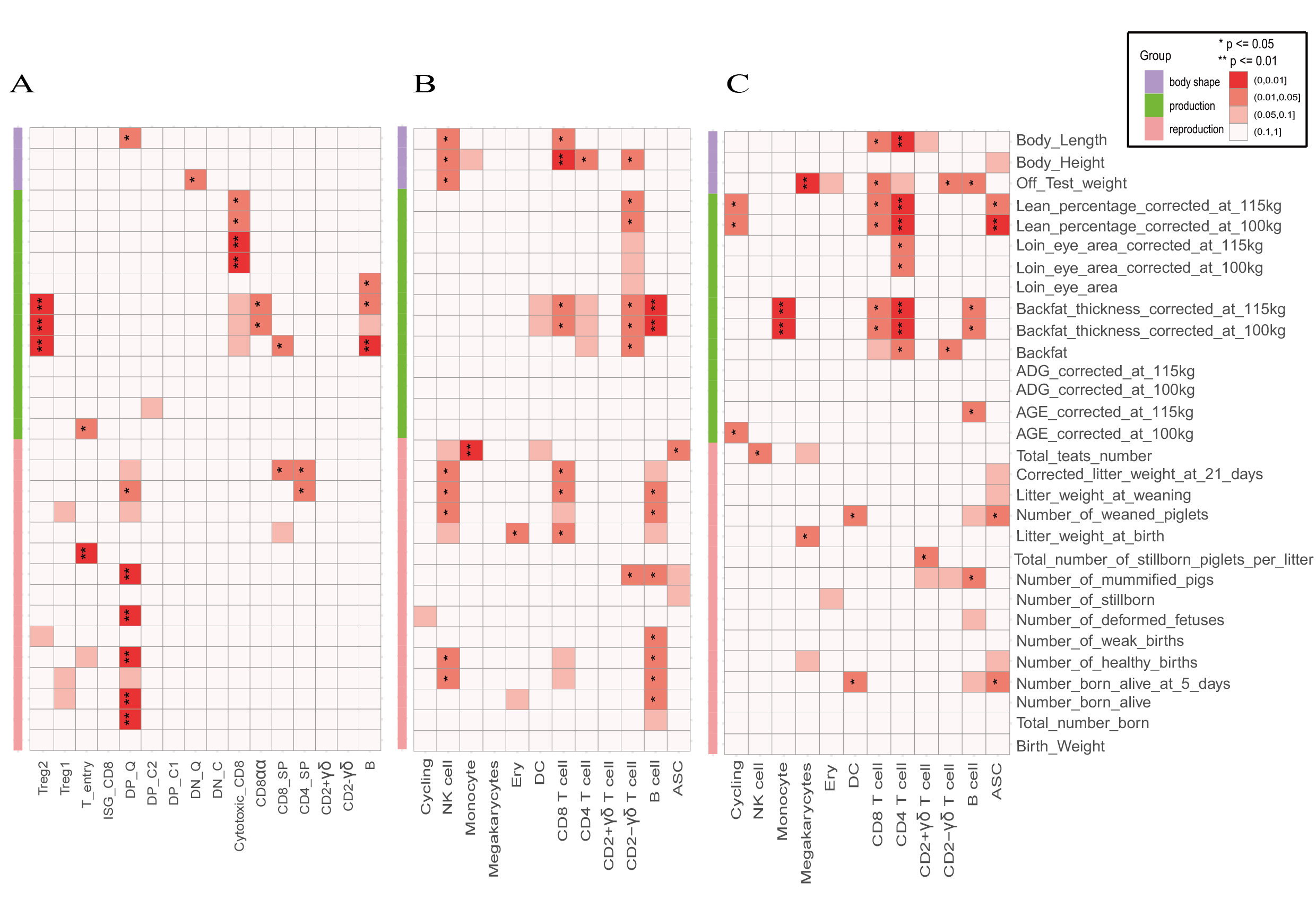

Supplement: Supplementary file 1 [file DataSheet_1.zip › Supplemental Figures/Supplementary Figure8.jpg]

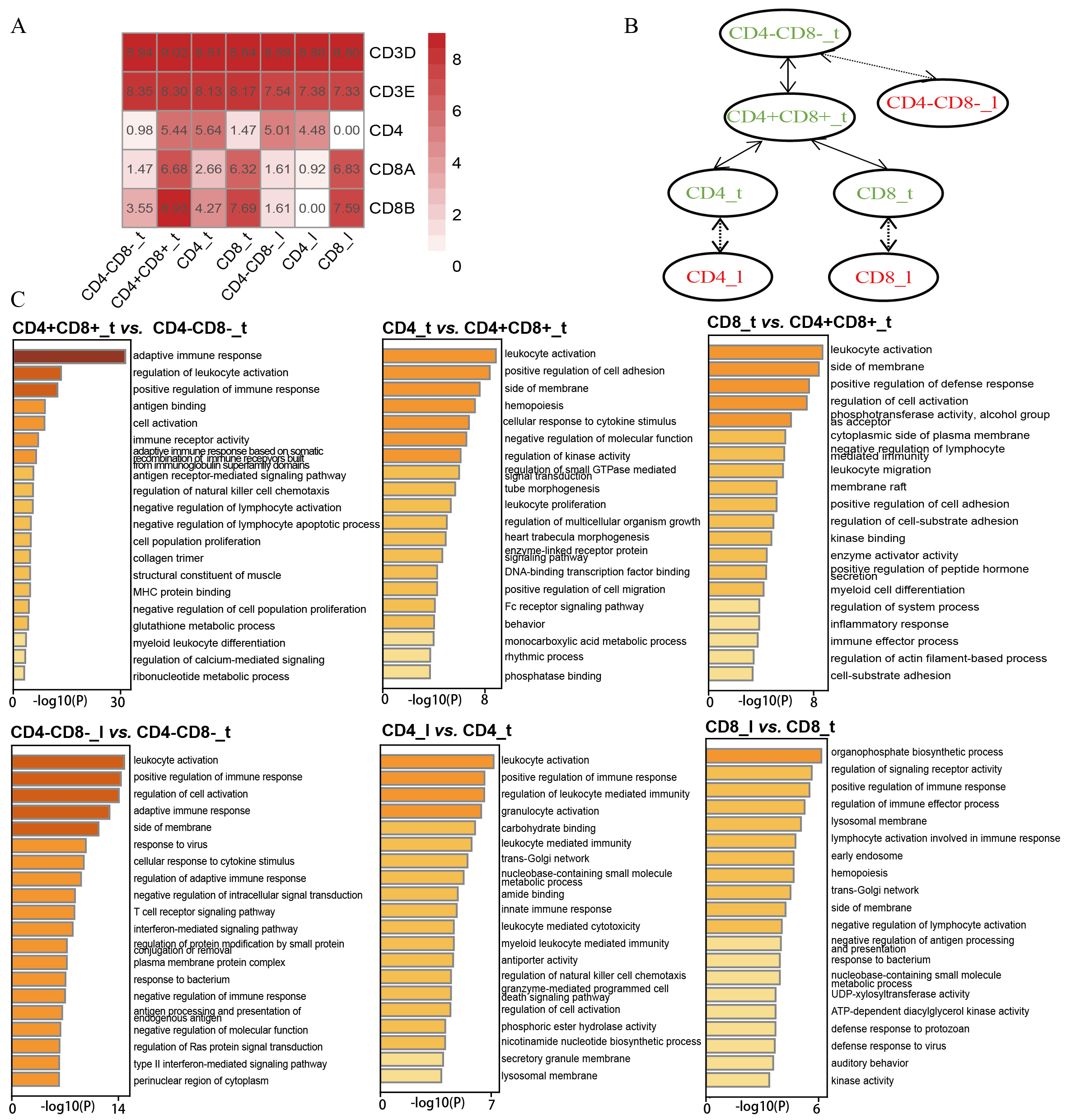

Supplement: Supplementary file 1 [file DataSheet_1.zip › Supplemental Figures/Supplementary Figure1.jpg]

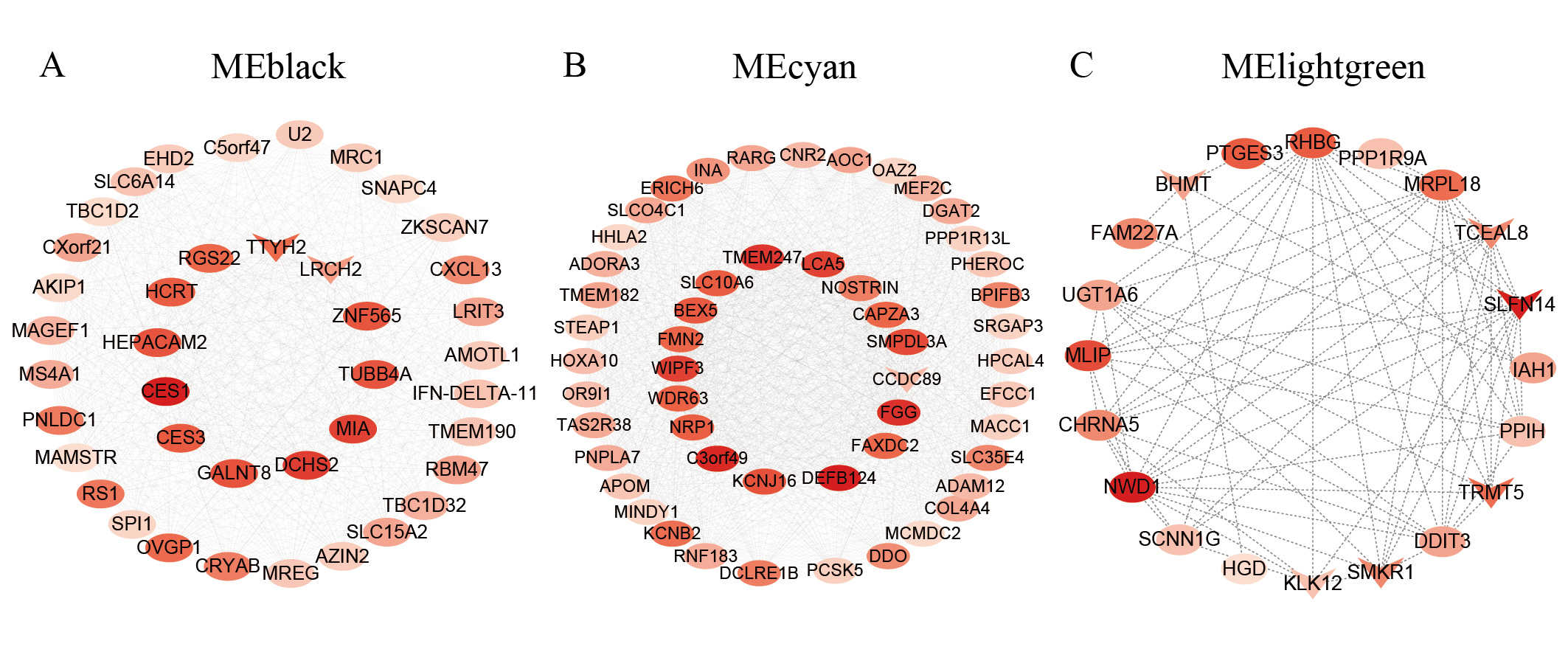

Supplement: Supplementary file 1 [file DataSheet_1.zip › Supplemental Figures/Supplementary Figure2.jpg]

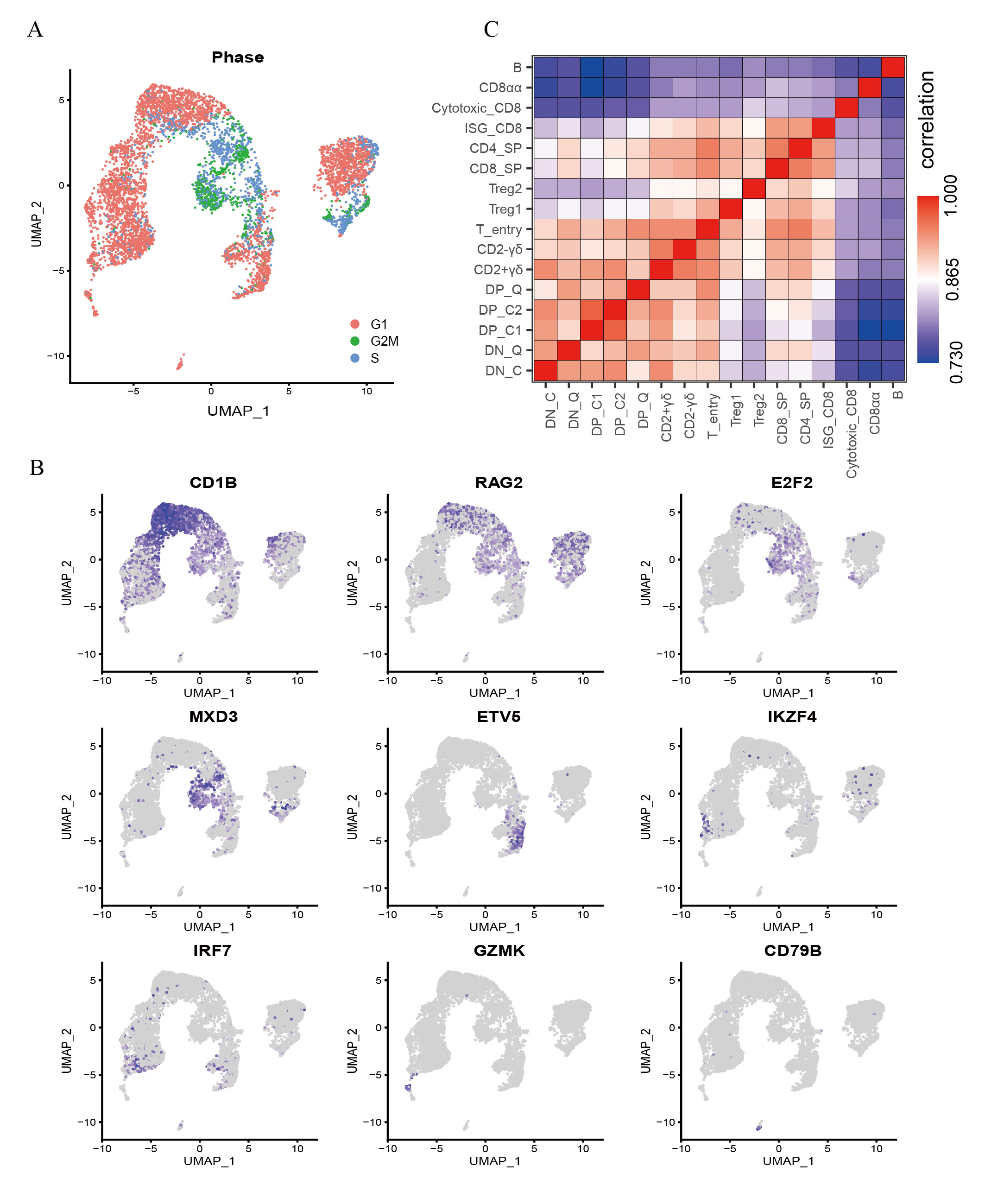

Supplement: Supplementary file 1 [file DataSheet_1.zip › Supplemental Figures/Supplementary Figure3.jpg]

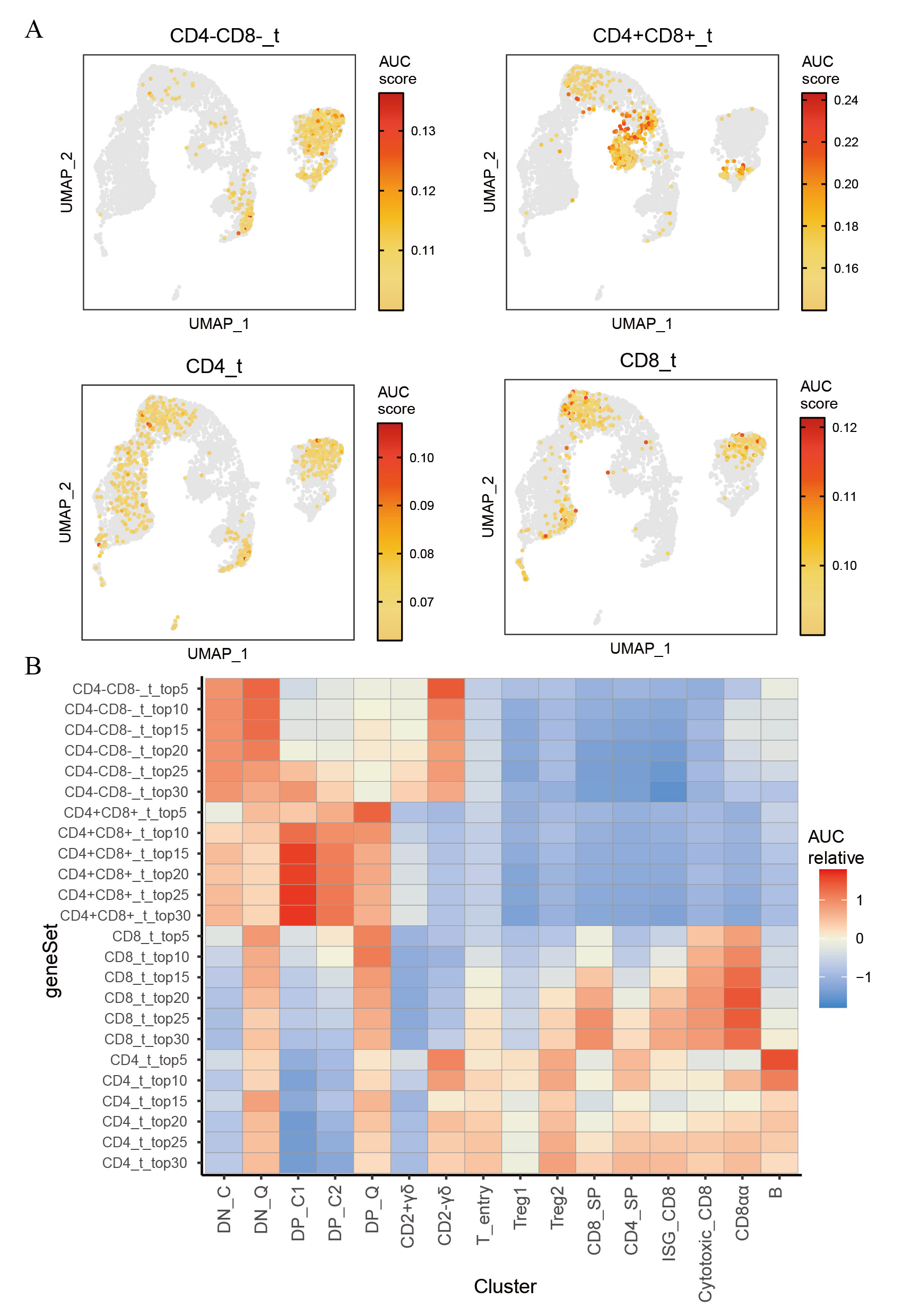

Supplement: Supplementary file 1 [file DataSheet_1.zip › Supplemental Figures/Supplementary Figure4.jpg]

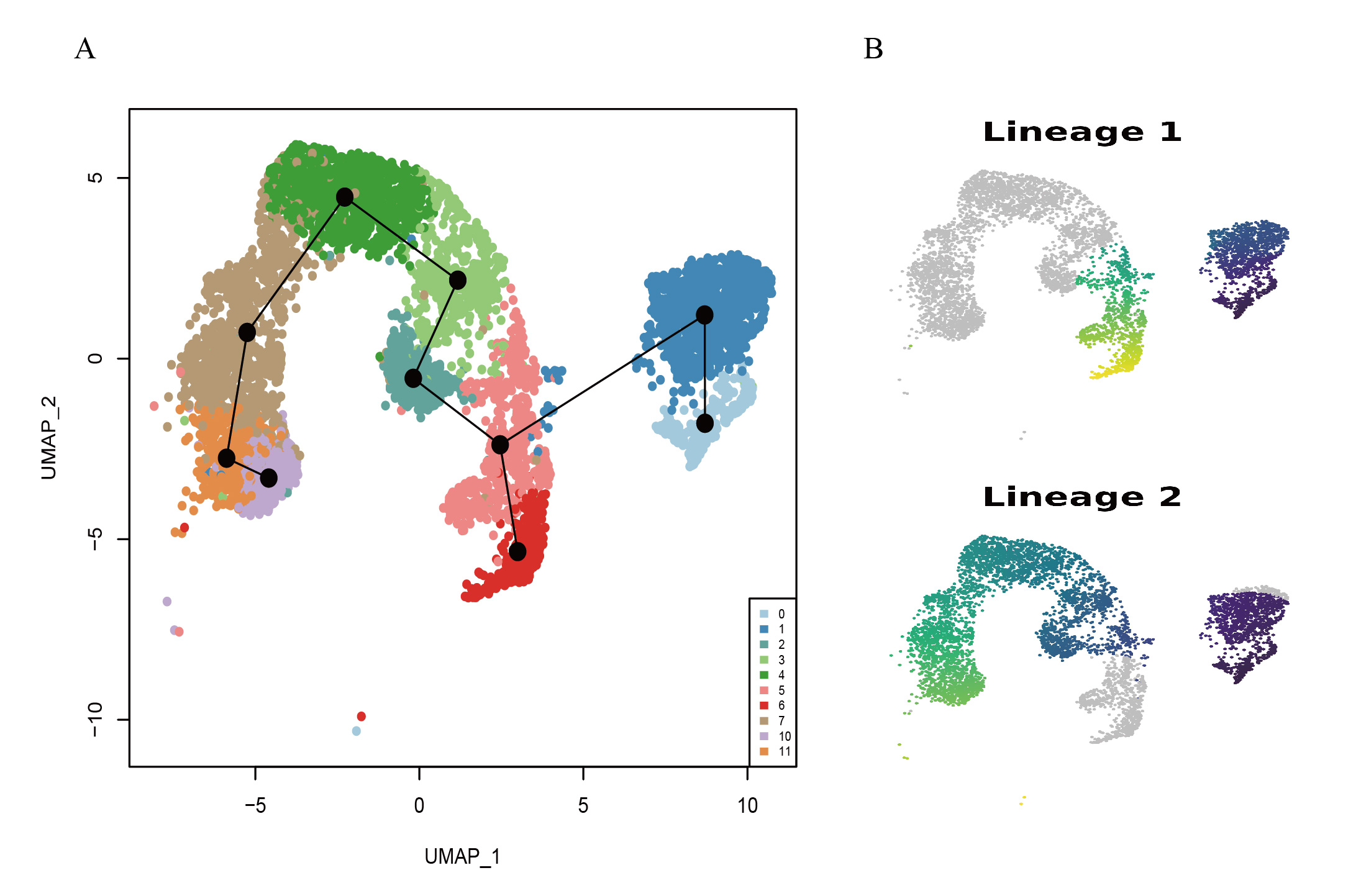

Supplement: Supplementary file 1 [file DataSheet_1.zip › Supplemental Figures/Supplementary Figure6.jpg]

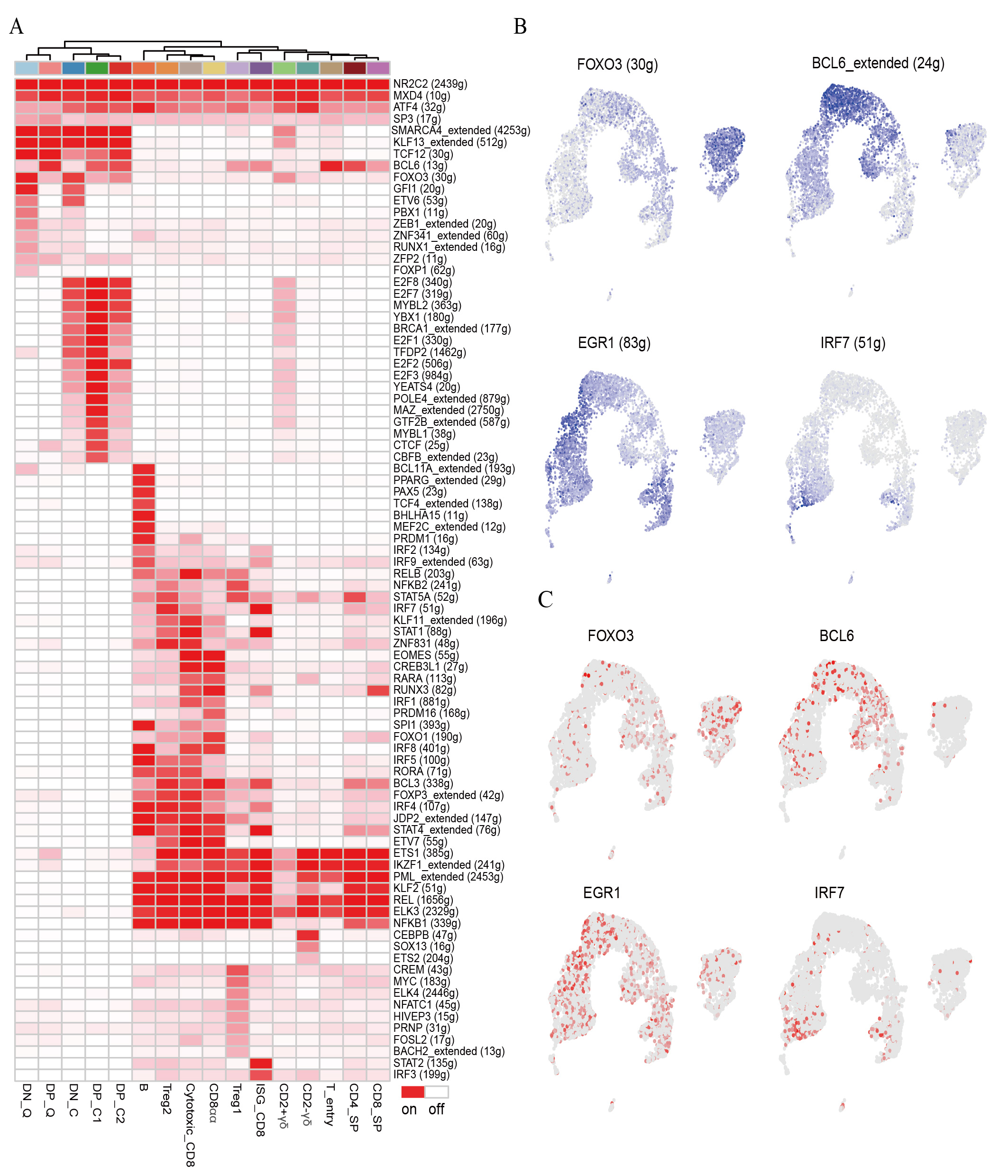

Supplement: Supplementary file 1 [file DataSheet_1.zip › Supplemental Figures/Supplementary Figure7.jpg]
